# High-Resolution Mapping of Gene Expression Using Association in an Outbred Mouse Stock

**DOI:** 10.1371/journal.pgen.1000149

**Published:** 2008-08-08

**Authors:** Anatole Ghazalpour, Sudheer Doss, Hyun Kang, Charles Farber, Ping-Zi Wen, Alec Brozell, Ruth Castellanos, Eleazar Eskin, Desmond J. Smith, Thomas A. Drake, Aldons J. Lusis

**Affiliations:** 1 Department of Human Genetics, University of California Los Angeles, Los Angeles, California, United States of America; 2 Department of Computer Science, University of California Los Angeles, Los Angeles, California, United States of America; 3 Department of Medicine, David Geffen School of Medicine, University of California Los Angeles, Los Angeles, California, United States of America; 4 Department of Molecular and Medical Pharmacology, David Geffen School of Medicine, University of California Los Angeles, Los Angeles, California, United States of America; 5 Department of Pathology and Laboratory Medicine, University of California Los Angeles, Los Angeles, California, United States of America; 6 Molecular Biology Institute, University of California Los Angeles, Los Angeles, California, United States of America; 7 Department of Microbiology, Immunology, and Molecular Genetics, University of California Los Angeles, Los Angeles, California, United States of America; The University of Queensland, Australia

## Abstract

Quantitative trait locus (QTL) analysis is a powerful tool for mapping genes for complex traits in mice, but its utility is limited by poor resolution. A promising mapping approach is association analysis in outbred stocks or different inbred strains. As a proof of concept for the association approach, we applied whole-genome association analysis to hepatic gene expression traits in an outbred mouse population, the MF1 stock, and replicated expression QTL (eQTL) identified in previous studies of F2 intercross mice. We found that the mapping resolution of these eQTL was significantly greater in the outbred population. Through an example, we also showed how this precise mapping can be used to resolve previously identified loci (in intercross studies), which affect many different transcript levels (known as eQTL “hotspots”), into distinct regions. Our results also highlight the importance of correcting for population structure in whole-genome association studies in the outbred stock.

## Author Summary

In rodents, as in humans, traits such as obesity or diabetes are under the influence of many genes spread throughout the genome. Using linkage analysis, the locations of the major contributing genes can be mapped only to very large regions of chromosomes, usually encompassing hundreds of genes. This has made it difficult to identify the underlying genes and mutations. Another approach, analogous to genome-wide association in human populations, is to use association analyses among outbred stocks of mice. In this proof-of-principle article, we make use of common variations that locally perturb gene expression to demonstrate the greatly improved mapping resolution of association in mice. Our results indicate that association analyses in mice are a powerful approach to the dissection of complex traits and their underlying molecular networks.

## Introduction

Quantitative trait locus (QTL) analysis has been the primary tool for geneticists to study complex genetic traits in experimental organisms. However, while such QTL mapping has great power to identify loci controlling the traits, resolution of mapping is usually quite low and as a result few candidate genes have been successfully identified using this approach. The use of molecular phenotypes, in particular gene expression levels, as quantitative traits for mapping, coupled with the ability to measure thousands of such traits simultaneously, has added a tremendous spark to the field of complex trait genetics. The integration of expression QTL (eQTL) with complex clinical traits using statistical modeling has allowed the identification of genes and pathways involved in a variety of complex traits. Some of the recent successes of this integrative approach have been identification of causal genes underlying the QTL for clinically relevant trait [1]–[3], the identification of genomic loci regulating the expression of biological pathway genes[4], the identification of genomic hotspots harboring master regulators [5]–[7], and prioritization of candidate genes underlying physiological trait QTLs [8]. Moreover, mathematical models have been developed to construct gene expression networks [9],[10], deduce the causal relationship between different components of the network [11], and understand the transcriptional regulation of the genes [12].

Despite these successes, such integrative genomic approaches using F2 populations suffer from the same limitation that has hindered the success of the traditional physiological trait QTL mapping, namely lack of resolution in mapping [13]. To overcome the lack of resolution problem, Flint and colleagues recently investigated the use of outbred stocks of mice to simultaneously detect and fine map physiological trait QTLs [14]–[16]. In the first of the two recent studies, they used 790 outbred mice (MF1) to study the genetics of behavioral traits and successfully mapped three QTLs within a 1cM region [14]. In the second study, the authors extended this approach to multiple traits and mapped 97 metabolic and human disease related phenotypes to intervals of 2.8 Mb (average 95% confidence interval) by using over 2000 heterogeneous stock mice [15]. The success of these studies prompted us to investigate the potential use of outbred mice for eQTL studies, where many validated quantitative trait genes for expression traits have been identified. In this report, we present the results of a whole genome association study for the liver gene expression profiling of 110 MF1 mice and compare the results obtained in this population with previously published linkage studies in F2 mice [17].

## Results

A total of 110 outbred MF1 mice were studied for whole genome transcript levels in liver and subjected to genotyping using the Affymetrix 5K Mouse Chip. From the 5024 SNPs on this array, 1813 SNPs (about one third of total SNPs) had a minor allele frequency of 5% or greater, and were used for the analyses described below. The average and median distance between neighboring SNPs for these 1813 markers were 1.38 Mb and 0.57 Mb respectively. To determine the percent coverage of the genome by these 1813 SNPs, we used the average distance of the adjacent markers which had an r squared value of 0.9 or higher. This analysis showed that on average adjacent markers will have such a high LD when within 1.03Mb. Based on this, approximately 3000 informative evenly spaced markers are needed to allow coverage of the entire genome. This calculation is based on the assumption that the haplotype blocks in this population are all about the same size. However, as we show below, this is not true for some regions of the genome, and thus the estimate is very approximate. Given the non-uniform distribution of the 1813 markers across the genome the genetic coverage for these set of markers is 72% of the genome. This means that in the whole genome association analysis described in this report we would expect to miss 28% of the signals.

Gene expression measurements were performed on RNA isolated from liver using Illumina's mouse whole genome expression BeadChip (MouseRef-8-v1 Expression BeadChip) (see Materials and Methods). We applied two filtering criteria to the 24048 probes on the microarray. The first filtering criteria was based on the detection p-value calculated for each probe (see Materials and Methods). This filtering step eliminated any probe with low signal which could be due to nonspecific hybridization. The second filtering criterion was based on the recent report by Walter et al [18] which they showed that for the Affymetrix platform the presence of SNP within the 25mer probe sequence may affect the hybridization of transcripts and lead to artifactual detection of local (cis) eQTL. To investigate if this applies to the 50mer probe sequences of the Illumina microarrays used in the current study, we examined the degree of enrichment of SNPs in probes with local eQTL vs probes with no local eQTL. We found that from the 10765 probes with reliable signal and unique genomic location 602 probes had a local eQTL and from these 105 contained at least one SNP (as determined from the Perlegen SNP database) within their probe sequences (17%). In contrast, from the 10163 probes with no evidence for local eQTL 647 (6%) contained one or more annotated SNPs. This means that the proportion of probes with SNPs in the sequence is significantly higher for probes determined to have local eQTL vs probes with no local eQTL (chi squared statistics p-value for such enrichment was <10^−16^). These results suggest that, as with Affymetrix arrays [18], the presence of SNP within the probe sequence on the Illumina microarray might result in artifactual local eQTL detection. Therefore, to overcome such a bias we excluded any probe with one or more SNPs in its sequence. The two filtering criteria combined yielded 10013 probes (from the original 24048) which we used as a starting set for the whole genome association analyses described below.

**Figure 1:**
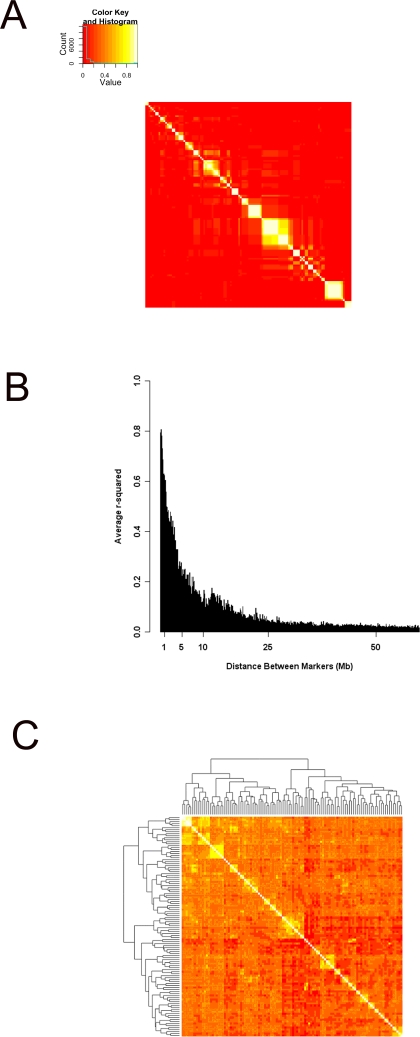
LD and population structure in the MF1 population. Panel (A) shows the LD structure on chromosome 1. The order of markers in the heat map follows the physical location of the marker along the chromosome with the most proximal starting at the bottom and on the right and the most distal marker on the top and on the left. The correspondence between color and r^2^ is shown in the insert. Panel (B) shows the distribution of r^2^ values between markers located at various distance from each other. Each bar depicts a 100 kb bin for the distance between marker pairs. The average r^2^ for marker pairs within 2Mb of each other is 0.58, for markers between 2 to 5 Mb is 0.3, and for markers 5Mb or more away from each other is 0.05. Panel (C) shows the heatmap visualization of genetic similarity between individual mice. The dendograms on the top and on the side of the heat map are based on the hierarchical clustering of genome wide genotype similarity of the 110 MF1 mice.

We first computed the degree of linkage disequilibrium in the population of 110 MF1 mice. Pair-wise r^2^ were calculated among all the SNPs, and the average r^2^ measures for different ranges were used to look at the LD structure in the population. Visual inspection of the LD between markers within the same chromosome revealed a complex LD structure in the population (Figure 1A and Figure S1). In particular, it was evident that the extent of LD varied in different regions of the genome. Moreover, although for most regions highest LD was between adjacent markers, in some cases non-adjacent markers showed a higher LD than adjacent ones. To quantify the extent of LD between the markers in this population, we created 100 kb bins of various distances between marker pairs and calculated the average r^2^ for each bin. As shown in Figure 1B, the average r^2^ dropped with increasing distance between markers. The average r^2^ for markers within 2 Mb of each other was 0.58, for markers between 2 to 5 Mb was 0.30, and for markers 5Mb or more apart was 0.04, suggesting that the extensive LD exists over several Mb. For markers located on different chromosomes, the LD was low (the mean r^2^ was 0.015 and the median was 0.008). Despite this low r^2^, inspection of the distribution of the chi-squared statistics p-values for expected r^2^ in absence of LD indicated significant LD between certain pairs. These observations were consistent with our visual inspection of LD maps suggesting the existence of a complex relationship pattern among different loci, presumably due to population substructure within the MF1 stock.

To further investigate this we performed hierarchical clustering of mice based on the kinship matrix which we derived from the overall correlation of genotypes between pairs of mice. The results revealed clear evidence of familial clustering (Figure 1C) indicating differences in relatedness. In addition, several multi-leveled larger clusters were observed with weaker levels of similarity, suggesting a complex genetic relatedness between the samples. The potential confounding effect from population structure was further supported by the fact that a very large number of expression levels are significantly explained by genetic relatedness between individuals. Using variance component test and at the 5% FDR level, 30.2% (3027) of transcripts were significantly associated with genetic background while only 1.5% (151) are expected by chance at the same threshold. In addition, for 19.8% (1985) of transcripts, more than 50% of variance was explained solely by the genetic background effect. This indicated that correcting for population structure is essential to avoid larger numbers of false positives.

In order to correct for population structure we used Efficient Mixed Model Association (EMMA). The underlying statistical algorithm for performing such correction has recently been published [18] and is briefly explained in the Materials and Methods section. In summary, EMMA controls for population structure and familial relatedness by modeling the gene expression on two terms (plus the random error term): one is the SNP genotype and the other is a term which takes into account the population structure. This term, which is estimated based on the genetic similarity of mice in the population, essentially captures the variance attributable to population structure and provides a better estimate of SNP effect and its significance on gene expression. Without partitioning this term, the variance due to the genetic structure will be falsely attributed to the SNP and might result in a false positive association signal. To examine the effects of correcting for familial structure on the genome wide association, we compared the results of the association before and after correcting for population structure using a linear additive model and a linear mixed model. Table 1 shows the results for various FDR thresholds and for both local (primarily *cis*) and distant (*trans*) eQTL. Before correction, regression analysis of gene expression on markers revealed a total of 812 significant associations (at FDR of 1%, corresponding to the p-val of 2.29e-06). From these, 444 (55%) were local and 368 (45%) were distant eQTL. After correcting for population structure, there were about two thirds as many significant associations as found originally (478 significant associations at the FDR of 1% corresponding to the p-value of 1.05e-06). This result suggested that about one third of the associations found in the absence of correction were false positives due to the relatedness of the mice. The reduction in significant associations among local and distant eQTL, however, was not the same. For local eQTL, there were 18% fewer associations after correction (366 vs 444), but for the distant eQTL there was a 70% reduction in the number of associations found (112 vs 368). We also examined the results at 5% and 10% FDR thresholds (Table 1) and as with the 1% FDR, we observed a similar pattern where the total number of associations was less after correction and distant eQTL were more affected by this correction than local eQTL. This inflation of p-values resulting from the population structure was also evident from the pattern and number of significant p-values for each transcript (Figure S2).

**Table 1:** Comparison of local and distant eQTL before and after correction for population structure.

%FDR	Local eQTL			Distant eQTL		
	Uncorrected	Corrected	% change	Uncorrected	Corrected	% change
1	444	366	18	368	112	70
5	569	446	22	1867	334	82
10	668	492	26	4616	704	85

**Figure 2:**
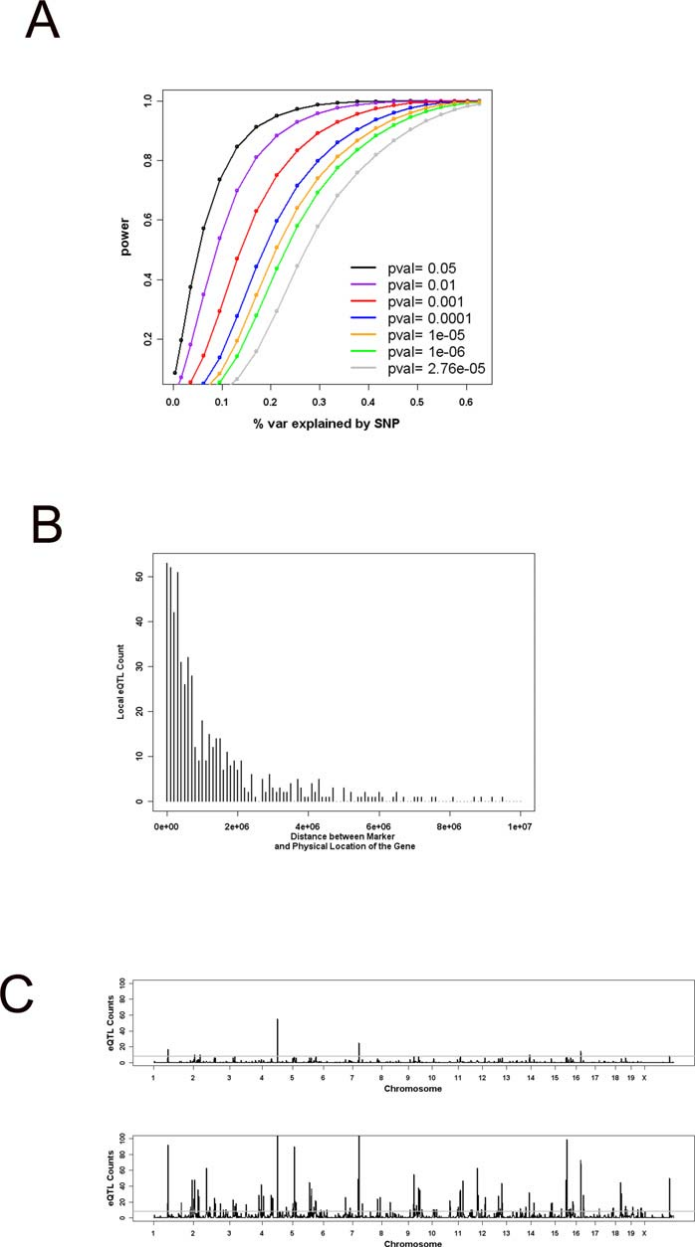
Power analysis and the genetic architecture of local and distant eQTL. Panel (A) shows the power calculation performed for genetic background effect of 0.3. Various curve colors represent the power associated with various p-value cutoffs. (grey = 0.05, green = 0.01, orange = 0.001, blue = 0.0001, red = 1e-05, purple = 1e-06, black = 2.76e-05 which is equivalent to the Bonferroni correction). For each calculation, the minor allele frequency is assumed 0.3. Panel (B) shows the distance of the association peak marker from the physical location of the gene for local eQTL identified in the MF1 population. Panel (C) shows the distant eQTL hotspots across the genome before (bottom) and after (top) population correction. The genome is represented as 1287 equally sized bins of 2 Mb. The gray line depicts the 0.05 genome wide significance for eQTL enrichment after Bonferroni correction (p-value of 3.9e-05).

One of the limitations of using outbred stock for mapping complex traits has been the statistical power issue and the need to include large number of mice in the study [13]. In addition, the presence of population structure between the animals can also have a negative impact on the statistical power. To assess the power of the current study, we performed power calculations under various genetic background (population structure) effects (Figure 2A and Figure S3). As shown in Figure 2A, for minor allele frequency of 0.3, the average minor allele frequency in our population, and the genetic background effect of 0.3 at the 10% FDR level (p-value = e-05), this study has over 60% power to detect QTL typical of what is expected from local eQTL (30% variance explained) as estimated from intercross data (unpublished data). For distant eQTL, however, where the effects are smaller (typically less than 10% of variance explained) at the same FDR level the use of 110 related mice will have relatively small power (<20%). These results imply that for eQTL described below the local eQTL detected reflect the majority of true local eQTL present in the population and for distant eQTL there may be a significant number associated with type I and/or type II errors.

We next examined the eQTL structure in the MF1 mice. As shown in Table 1 (and Table S1), 1196 eQTL had significant association at the 10% FDR (p-value of 2.43e-05) after correcting for population structure, which greatly exceeded the 119 expected by chance. Among the 1196 eQTL, 24 were due to different probes of the same gene mapping to the same location. This reduced the number of unique eQTL for each gene to 1172. From these, 471 were classified as local or *cis* eQTL (defined as the association peak marker for transcript of a gene mapping to within 10Mb of the physical location of the gene itself) and 701 were classified as distant or *trans* eQTL (The significant results for the whole genome association analysis at various FDRs can be found in Table S1 and the top 10 most significant distant eQTL at 10% FDR are shown in Table 2). From the 1172 eQTL, which belonged to 1093 genes, there were a total of 1019 single gene associations, 69 genes had two associations, and 5 genes had more than two associations. From the 69 genes with two associations, 27 genes had one local and one distant eQTL and 42 genes had two distant eQTL. In general, the p-values for local eQTL (mean–log of p-value = 11.5) were more significant than the p-values for distant eQTL (mean–log of p-value = 5.4). Local eQTL are very likely to be due to a variation either within the gene or in the regulatory region in close proximity to the gene. To investigate the resolution achieved in the MF1 data we calculated the distance of the association peak marker to the physical location of the gene for each local eQTL. This analysis revealed that the median distance of the peak markers from the physical location of the gene was 0.67 Mb and for 25% of the genes the peak marker was located less than 300 Kb away from the gene itself (Figure 2B).

**Table 2:** Top 10 most significant associations for distant eQTL.

Gene Symbol	Chromosomal Gene Location	Mapping SNP	SNP Chromosome	SNP Position (Mb)	Association p-value
*Lrp11*	10	rs13478347	5	80.5	1.61E-29
*Mat2b*	11	rs13477797	4	78.6	4.35E-10
*Cyp2c54*	19	rs13479573	7	127.1	6.78E-10
*9330164H19Rik*	7	rs6164040	4	79.8	4.04E-09
*2810027O19Rik*	2	rs13475914	1	71.5	4.34E-09
*Kif3a*	11	rs13479070	6	137.9	1.09E-08
*Vps33b*	7	rs6351643	2	18.7	1.13E-08
*Birc4*	20	rs3724460	2	104	1.48E-08
*Pcolce2*	9	rs13478096	5	4.99	1.53E-08
*Sgk*	10	rs13475914	1	71.5	1.64E-08

We also searched for the presence of co-localizing distant eQTL (eQTL “hotspots”). To do this, the entire genome was divided into 2 Mb bins (1287 total bins) and the number of significant distant eQTL were counted in each bin. Plotting of the eQTL frequencies at various genomic regions indicated a non-random distribution of the mapping locations (Figure 2C). Several ‘hotspots’ were identified, with the most highly enriched loci on Chromosome 1 (17 eQTL), Chromosome 4 (55 eQTL), Chromosome 7 (25 eQTL), and Chromosome 16 (15 eQTL). To assess the validity of these hotspots, we randomly grouped mice into two subsets and reanalyzed each subset for the presence of co-localizing distant eQTL. We repeated this procedure four times to test for the preservation of these four highly enriched hotspots in each of the subsets (Figure S4). From these four hotspots, the Chromosome 4 hotspot was present in 7 of the 8 subsets created. Chromosomes 1 was replicated 4 times, Chromosomes 7 replicated three times, and Chromosome 16 replicated twice in the subsets analyzed. For Chromosomes 1, 7, and 16, the inconsistency in replication could either be due to an artifact of population structure not accounted for by our correction method [20] or to lack of power resulting from doing the analysis on half as many animals as in the original analysis. The presence of hotspots is consistent with the notion that the causal genetic variant is located within a master regulator of gene expression for group of genes. To identify candidate master regulator genes for each of these hotspots, we searched for local eQTL within each region. On Chromosome 1 we found no local eQTL; on Chromosome 16 we found one local eQTL homogentisate 1, 2-dioxygenase (*Hgd*); on Chromosome 7 we found 6 local eQTL including the enhancer binding protein CCAAT/enhancer binding protein alpha (*Cebpa*), lipolysis stimulated lipoprotein receptor (*Lisch7*), peptidase D (*Pep4*), coiled-coil domain containing 123 (*Ccdc123* or *2610507L03Rik*), androgen regulated gene RP2 (*Nudt19* or *D7Rp2e*), a Rho GTPase binding protein rhophilin 2 (*Rhpn2*); and in the most enriched hotspot on Chromosome 4 we only found one local eQTL methylthioadenosine phosphorylase (*Mtap*).

Since MF1s offer a higher mapping resolution, this resource may be used to fine map eQTL previously identified in other crosses. The limitation is that not all the eQTL found previously will be present in the MF1 population. Here we sought to compare the mapping results in the MF1 population by empirically estimating the fraction of eQTL detected in this study compared to what was found in a previously published cross from our laboratory. For this comparison we used the previously reported eQTL study of the liver tissue for the F2 intercross population generated between C57BL/6J.ApoE^−/−^ and C3H/HeJ.ApoE^−/−^ parental strains (herein referred to as the BxH cross) [17]. In order to make a direct comparison we used the Entrez-Gene accession IDs to map the probes across the Illumina and the Agilent microarrays. From the 10013 probes used in the MF1 genome wide association analysis 8437 had unique Entrez-Gene IDs, 8036 of which were also represented by one or more probes on the Agilent microarrays used in the BxH cross. Using the genome-wide suggestive LOD score of 3.5, a total of 8111 eQTL were present in the BxH study. From these, 1905 eQTL mapped to within 10Mb of the physical location of the gene and were classified as local eQTL and the remainder (6206) as distant. Intersection of the local eQTL for the common set of genes in the two studies (1905 eQTL in BxH vs 471 eQTL in the MF1) identified 163 genes. This amounts to ∼35% of the total local eQTL found in the MF1 study (163/471). Intersection of distant eQTL, however, gave a much smaller overlap. From the 760 distant eQTL in the MF1 there were only 9 present in the BxH data (7 expected by chance, P = 0.22) which is about ∼1% of the distant eQTL found in the MF1 study (Table 3). As discussed previously, the MF1 data has a low statistical power to detect distant eQTL especially at higher p-value cutoffs such as the one we used to compare the two datasets (P = e-05). Therefore, the lack of overlap between the distant eQTL in the MF1 data and the BxH data can be attributed to both the lack of power associated with detecting distant eQTL in the MF1 study and the conservative p-value cutoff chosen to detect these eQTL. Lowering the cutoff value for significance to 1.14e-04 (25% FDR) identified 26 overlaps with the BxH data (24 expected by chance, P = 0.3) and lowering this cutoff further to a nominal p-value of 0.001 resulted in 163 distant eQTL overlap between the two studies (140 expected by chance, P = 0.008).

**Table 3:** Common distant eQTL identified in both the BxH F2 linkage cross and the MF1 association study.

Gene Symbol	BxH Mapping Chromosome (Location Mb)	LOD Score	MF1 Mapping Chromosome (Location Mb)	p-value
*Alas2*	4 (62.5)	3.71	4 (43.9)	4.62E-06
*Cyp1b1*	18 (52.6)	3.67	18 (40.1)	1.20E-05
*Ptgds*	7 (144.9)	3.85	7 (132.7)	1.23E-05
*AI586015*	15 (62.8)	3.75	15 (69.5)	9.12E-06
*S3-12*	6 (115.9)	25.01	6 (115.3)	9.55E-07
*Centd2*	7 (64.4)	8.65	7 (50.2)	1.12E-05
*Slc36a4*	5 (78.1)	5.38	5 (59.5)	9.23E-06
*Foxred1*	4 (86.9)	6.02	4 (79.8)	5.10E-06
*Lrp11*	5 (84.1)	5.23	5 (80.5)	8.13E-29

**Figure 3:**
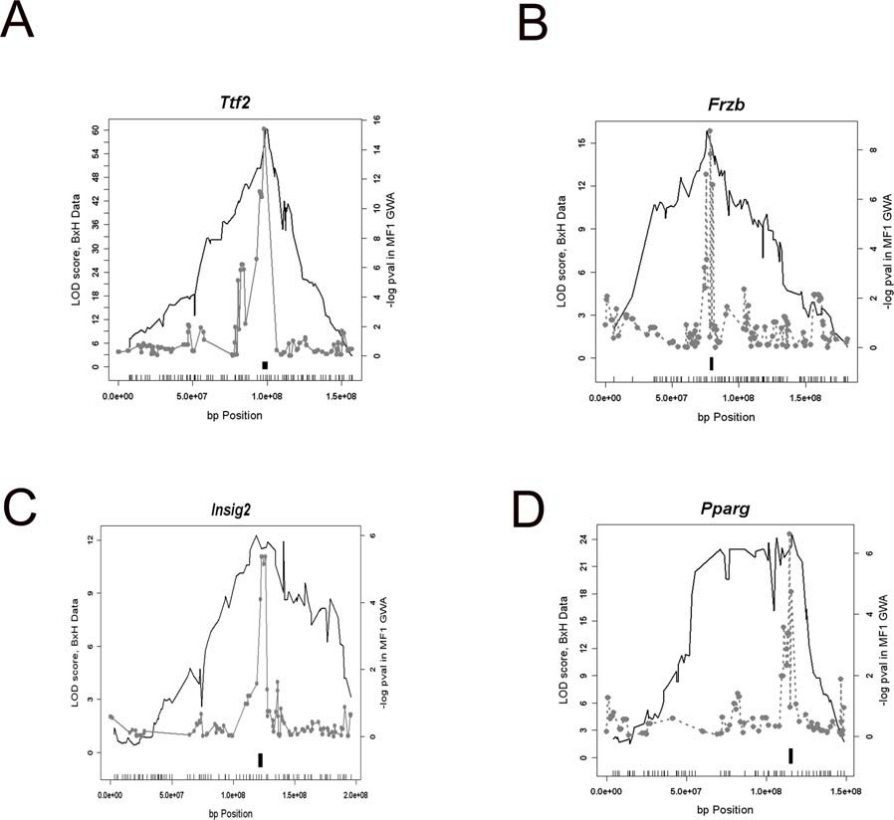
Comparison of four conserved local eQTL mapping results between the MF1 and BxH studies. In each plot, the black curve depicts the LOD curve in the BxH data, and the gray curve is the association result in the MF1 data. The physical location of the gene is shown by the black box. In each panel, the tick marks on x-axis depicts the physical location of the markers used in the BxH study. A) *Ttf2*, B) *Frzb*, C) *Insig2*, D) *Pparg*.

Previous studies suggest that outbred stocks offer a high resolution mapping resource, but these studies did not have prior knowledge of the location of the causal variant for the trait [16]. The presence of common local eQTL, where one can assume, with high confidence, that the causal genetic variant lies within or near by the physical location of the gene, between the BxH and the MF1 data sets provides a unique setting to directly demonstrate the higher mapping resolution attainable in the association study using MF1 outbred stock compared to the linkage study in the F2 population. Figure 3 illustrates the results of such a comparison for 4 shared local eQTL (*Ttf2, Insig2, Frzb, and Pparg*) between BxH and MF1 populations. As shown, the MF1 data (grey curve) provided much narrower candidate region than the BxH data (black curve). As expected for any local eQTL, the candidate regions in both studies encompassed the genomic region for the gene itself and peaked directly over the physical location of the gene. However, the QTLs in the BxH cross encompassed a much broader region than the association results in MF1 data. These results indicate that the MF1 population yields a much better resolution for eQTL mapping than a traditional cross.

**Figure 4:**
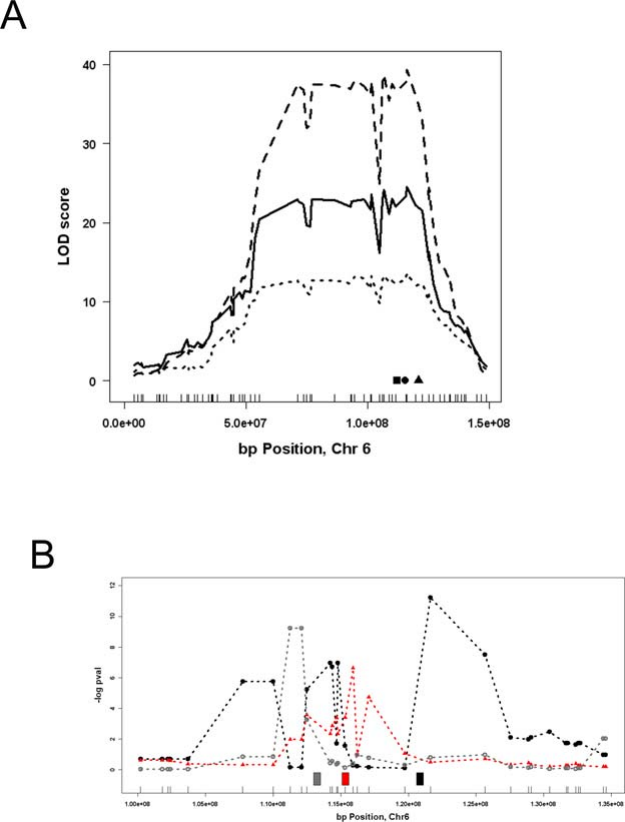
Comparison of the three conserved local eQTL on chromosome 6 between the BxH and MF1 studies. Panel (A) shows the BxH mapping results for *Pparg* (dashed curve), *Cidec* (solid curve), and *Bcl2l13* (dotted curve). The physical location of *Pparg* (circle), *Cidec* (square), and *Bcl2l13* (triangle) is shown at the bottom. Panel (B) shows the MF1 results for *Pparg* (solid triangles with dotted red curve), *Cidec* (open circles with gray dotted curve), and *Bcl2l13* (solid circles with dotted black curve). The physical location of the genes are depicted at the bottom with gray box for *Cidec*, red box for *Pparg*, and black box for *Bcl2l13*. Tick marks on x-axis depict the physical location of the markers in the BxH dataset.

We next turned to the distant (*trans*-acting) eQTL. Several eQTL studies with intercross or RI strain mice have observed that many distant eQTL map to the same location on a chromosome, giving rise to what is known as distant eQTL hotspots. Here we illustrate how the MF1 whole genome association analysis can be used to resolve such co-localization of distant eQTL. We had previously detected a distant eQTL hotspot in the middle region of chromosome 6. A total of 96 unique genes (99 probes) had a distant eQTL at this locus. Based on the assumption that the variation in the expression of the causal gene underlying this hotspot mediates its effects, we identified 8 local eQTL (Bcl2l13, Ogg1, Cidec, Atp2b2, Pparg, Clstn3, LOC380687, C1r) as primary candidate genes for this hotspot. Thus, judging by the F2 data alone, any one of these 8 local eQTL could be regulating any of the 96 distant eQTL. We turned to the MF1 data and asked, first, if we could resolve the co-localization of the local eQTL on the chromosome 6 locus and, second, if we could resolve the co-localization of the distant eQTL in this region. From the 8 local eQTL in the BxH F2 cross, 3 of them (*Bcl2l13, Pparg,* and *Cidec*) were also observed in the MF1 population. Figure 4 shows the mapping of these three local eQTL in the BxH and the MF1 populations. While the mapping results appeared indistinguishable in the BxH study (Figure 4A), association analysis in the MF1 successfully resolved these local eQTL and mapped them near the physical location of each gene (Figure 4B). Out of the three local eQTL, the local eQTL for *Cidec* mapped to two markers, rs13478971 and rs13478971, at 112.1 and 111.2 Mb, respectively, which are ∼1 and ∼2 Mb away from where the physical location of the *Cidec* gene (113.3 Mb). These were the closest markers to the physical location of *Cidec.* These results suggested the presence of either a distal regulatory element for this gene or the presence of a closely linked regulatory gene for *Cidec* on Chromosome 6. Genotyping with denser markers closer to *Cidec* location might correctly position the highest peak above the gene itself. Interestingly, the *Bcl2l13* transcript levels, in addition to mapping to the nearest marker to the physical location of the gene at 121.6 Mb, also showed significant association with markers located at 114.2, 114.3, and 114.7 Mb, suggesting the possible presence of an additional distal regulatory locus near the local eQTL for this gene. After resolving the three local eQTL, we examined the distant eQTL which mapped to this locus in the BxH cross and asked which of these distant eQTL mapped to any of these three local eQTL on Chromosome 6. From the 96 distant eQTL colocalizing to the chromosome 6 locus in the BxH study, 14 were replicated in the MF1 population (using the nominal p-value cutoff of 0.01). Judging by the location of the association peak markers, despite the co-localization in the BxH, these 14 eQTL mapped to varying loci within the chromosome 6 region. In particular, 3 of these genes (*S3-12, Calr3, Hmgcl*) mapped over the *Pparg* locus, another 2 genes (*Gpi1, Ctps*) mapped over the *Cidec* locus, and 9 genes had the most significant associations with markers at other than the three local eQTL loci (Figure S4). For the 5 genes mapping to either the Pparg or the Cidec loci we computed the 50 and 90 percent confidence (c.i.) intervals using bootstrapping (1000 sample sets). The S3-12 and Calr3 50% c.i. span a 1.3Mb region over the Pparg locus from 114.6Mb to 115.9Mb and the 50% c.i. for Hmgcl span a 1.9Mb region over the Pparg locus from 112.4Mb to 115.3Mb (the 90% c.i. for S3-12 was from 112.4Mb to 117Mb, for Calr3 was from 112.4Mb to 116.2Mb, and for Hmgcl was from 112.1Mb to 115.9Mb). For the two genes with the peak marker at the Cidec locus (Gpi1 and Ctps) the 50% c.i. were 3Mb (from 111.2Mb to 114.2Mb) and 1.2Mb (from 111.2Mb to 112.4Mb), respectively, overlapping with the Cidec locus. The 90% c.i. for these two genes were from 110Mb to 114.3Mb and from 111.2Mb to 114.6 Mb, respectively. It is noteworthy that *S3-12* is a known *Pparg* target gene [19]. These results show that the co-localization of eQTL in the BxH study can be successfully resolved with high resolution in the MF1 data.

**Figure 5:**
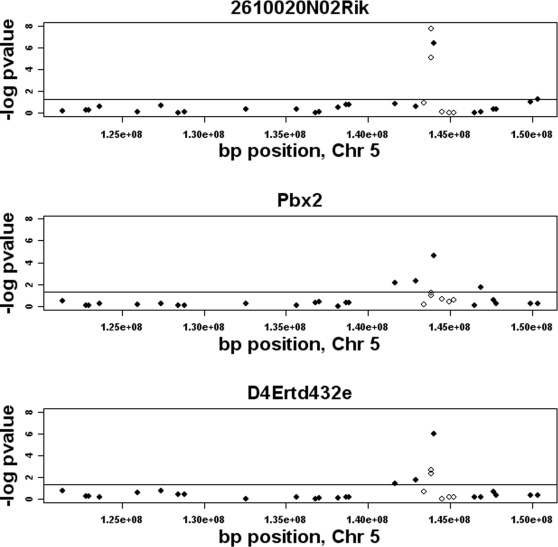
Fine mapping of the distal chromosome 5 locus. The three distant eQTL *2610020N02Rik* (top), *Pbx2* (middle), and *D4Ertd432e* (bottom) were fine mapped by typing additional markers in the region (open circles). Closed circles represent the original markers used in the whole genome association. The horizontal line corresponds to the nominal p-value of 0.05.

The amount of resolution achieved in our mapping study was limited to the density of the markers used (average marker density 1.37 Mb). Next, we asked whether typing more SNPs in a region would enhance the mapping resolution. For this, we focused on the distal locus of chromosome 5 where the results of the whole genome association had identified 3 distant eQTL (*Pbx2, 2610020N02Rik,* and *D4Ertd432e*) at the genome wide significance level of 2.43e-05 p-value (10% FDR). The candidate region for this association spanned a 3.6 Mb region (from rs13478570 at 142.8 Mb to rs13478583 at 146.4 Mb) with the peak marker at 144 Mb (rs13478573). To fine map this region each of the 110 animals were genotyped for an additional 5 markers by the PCR-ARMS technique [20]. The primers were designed such that they would be less than 500 kb away from the peak marker or each other (Materials and Methods). The fine mapping results are shown in Figure 5. For D4Ertd432e (bottom panel) and 2610020N02Rik (top panel) the fine mapping effort reduced the candidate locus to 1.1 Mb (located between rs29635622 at 143.4 Mb and rs32348286 at 144.5 Mb) containing 22 candidate genes. For Pbx2, the candidate region was reduced to 0.7 Mb interval between rs33492148 (at 143.8 Mb) and rs32348286 (at 144.5 Mb) containing 14 candidate genes. The 90% c.i. for these three genes (as determined by 1000 bootstrapping data sets) spanned a 500 kb region in the interval between 143.81Mb and 144.52Mb. These results suggest that MF1 population, with a relatively small sample size of 110, gives a sub-megabase resolution for mapping eQTL.

## Discussion

This report provides a “proof of concept” demonstration of the utility of genome wide association for the identification of genes contributing to complex traits in mice. A number of previous association studies with outbred stocks or different inbred strains have been reported, but these have in most cases not been validated since the underlying genes were not known [14], [15], [21]–[23]. We have taken advantage of many local eQTL that have been identified in a recent linkage study in mice to validate the association approach. We also have provided an overall view of LD structure in the MF1 population and have shown the importance of correcting for population structure in association analyses.

In this study we used both the local eQTL and the distant eQTL to investigate the attainable high resolution mapping of expression traits in the MF1 population. We used the local eQTL as a proof of concept because with high confidence we can predict where the genetic variant is located (i,e. near or within the physical location of the gene) [24]. Therefore, this allowed us to study the level of resolution one can achieve in the MF1 population. The comparative analysis of the four common local eQTL between the BxH F2 and the MF1 mice suggested that one can achieve a mapping resolution below 1 Mb. This is also evident from the fact that in all the local eQTL identified in the MF1 data about half the peak markers for the association mapped within 600 kb from the physical location of the gene. The sub-megabase resolution achieved for the eQTL is also supported by the fine mapping results for the distant eQTL for the chromosome 5 locus as well. These results are also comparable to the previous mapping studies for the behavioral traits in MF1 mice where the reported confidence intervals for 3 closely linked QTLs were between 250 to 750 kb [14]. Clearly, with larger numbers of mice and denser genotyping, we expect the mapping resolution in this population to increase and the confidence intervals to decrease.

The number of significant associations observed at various false discovery thresholds in this study (Table 1) were lower than the numbers reported in other genetical genomics studies [5],[6],[25],[26]. This was especially true for distant eQTL which, for most part, have relatively small effects. One of the reasons for this shortcoming is the lack of power associated with the small number of animals used in this study (110 mice). The presence of population structure, in turn, also negatively impacts the effective size of the animals used in the study. In fact, one of the limitations of using outbred stock for mapping complex traits has been the statistical power issue and the need to include large number of mice in the study [13]. Another important point related to the statistical power issue is the very stringent genome wide significant cut off chosen in the whole genome association analysis due to the multiple testing issue. Without doubt, the use of genome wide cut off value is the appropriate measure for screening significant associations across all the markers and all the gene expression traits in the genome (over 22 million total tests performed). However, in settings where the replication of previously found QTL is under investigation, the hypothesis to be tested is reduced to one trait and several markers along the previously mapped region. Therefore, there should be no need for selecting such a high cutoff value for significance. This was evident in our data when we attempted to resolve the *S3-12* and 13 other previously identified chromosome 6 locus distant eQTL. At the genome wide cutoff value, only 2 of these genes were significantly associated with markers on chromosome 6 locus, but with the nominal p-value of 0.01, 12 additional genes also showed evidence of significant association to this locus. The use of less stringent criteria for local QTL studies has also been implemented in other reports and shown to correctly rediscover and validate previously identified QTLs [27],[28].

Previous genetical genomics studies reported the presence of genomic hotspots where large groups of eQTL collectively map to single loci in the genome [4],[5],[29]. In the current study, we were also able to identify such hotspots in the MF1 population, but the number of co-localizing eQTL within each hotspot identified in our study is considerably less than what has been reported before in other crosses [5],[29]. This is partly due to the lack of power to detect distant eQTL (as mentioned above) and partly due to the high resolution mapping achieved in the MF1 population. In general, the presence of eQTL hotspots indicates either the presence of a master regulator gene which regulates the expression of group of genes together or the presence of several tightly linked genes within the hotspot, each of which regulates the expression of subset of the genes which map to this locus [30]. In the case of an F2 population, since the mice carry relatively few recombinations, these two alternatives appear alike and indistinguishable from the mapping data. In the MF1 data, however, since the genome is finely grained, one can resolve a hotspot due to multiple linked genetic variants into its individual components. We used the chromosome 6 locus as an example and were able to resolve the 14 distant eQTL into several groups based on which marker they associated with most strongly. Among these, the group that showed significant association with the Pparg locus comprised three genes one of which (S3-12) has been associated with Pparg gene previously [19],[31] and another gene (Hmgcl) has been shown to be coregulated with Pparg at the transcript level by thyroid hormone [31].

It has been widely acknowledged that standard statistical tests that do not account for population structure or familial relatedness are prone to identify spurious associations [32]–[34]. Different levels of molecular variance between different pairs of individuals are likely to induce different levels of polygenic background effects, invalidating the independence assumption of standard statistical tests such as t-test or ANOVA. Recent studies illustrate that the linear mixed model effectively captures confounding effects due to heterogeneous genetic relatedness [35],[36] more effectively than previous approaches such as Structured Association [37], Principal Component Analysis [38] or Genomic Control [39]. In this report we provide additional evidence for how failure to correct for such population structure can result in many more false positive associations. We also show that distant eQTL are more prone to such artifactual associations due to their relatively small effect sizes and higher likelihood of being false positives.

Genetical genomics is becoming increasingly popular due to its promise to bridge the gap between the physiological traits and the genetic variations in the population. To fully take advantage of such an approach it is imperative to understand the nature of the association between the transcript level of the gene and the causal genetic variation. Molecular networks underlying the physiological traits cannot be properly constructed without the proper knowledge of the interaction among genes. We believe that the genetical genomics approach coupled with the high precision mapping offered by the MF1 outbred stock will significantly advance the potential for identifying regulatory genes for distant eQTL and provide the necessary components to build such biological networks.

## Materials and Methods

### Animals

Female MF1 mice, approximately 4–6 weeks of age, were purchased from Harlan (Indianapolis, Indiana, USA). These animals were fed Purina Chow (Ralston-Purina Co., St. Louise, MO) containing 4% fat until 19 weeks of age, and then fed a Western diet (Teklad 88137, Harlan Teklad, Madison WI) containing 42% fat and 0.15% cholesterol for ∼14 weeks until they were sacrificed at 33 weeks of age. All mice were maintained on a 12h light/dark cycle. Mice were fasted for 5 hours before being euthanized.

### Genotyping

For the initial genotyping, the Affymetrix GeneChip Mouse Mapping 5K SNP platform was utilized. The DNA used for the genotyping was isolated from the tail clips of each mouse using the Qiagen's DNeasy tissue kit (cat# 69506). Overall, a total of 5024 SNPs were genotyped. The genomic location of all the analyzed SNPs were based on the snpdb126 (http://www.ncbi.nlm.nih.gov/SNP/index.html).

Fine mapping on the distal region of Chromosome 5 was performed using the PCR-ARMS technique [20]. A total of 6 SNP markers were selected for fine mapping: rs29635622 at 143400193 bp position, rs33318740 at 143817829 bp position, rs33492148 at 143854676 bp position, rs32348286 at 144521447 bp position, rs29524465 at 144918013 bp position, and rs33719947 at 145196793 bp position. These primers were chosen so that the distance between adjacent markers did not exceed 500kb. To carry the PCR-ARMS a set of tetra-primers were designed using the http://cedar.genetics.soton.ac.uk/public_html/primer1.html website [20] for each marker. The tetra-primer sequences of each marker and the expected band size for each are as follows: 1) rs29635622 forward inner primer (C allele specific) GCTTATTTGCATACTTTGCGATGTAGAC, reverse inner primer (T allele specific) AACTATCCAAATGCACACTGAAGCCA, forward outer primer GTGCTATCTCTTCAGCCCAGAGTGATAT, reverse out primer GAGGAGCGAACCATTCTCTAAAAGTTGT, C-allele size = 133bp, T-allele size = 163 bp, outer-products = 242 bp; 2) rs33318740 forward inner primer (A allele specific) AAGATGCCGGCCCCAGATTGCCCTGTGA, reverse inner primer (G allele specific) GGGAGAAAGCTCCCTGCTTTGTCCAAACTC, forward outer primer GGAAGGTGAGGAGACAGGCTTCCGGCAG, reverse out primer CTTATGGCAAACCACCCTGCCCAGCAGA, A-allele size = 193 bp, G-allele size = 162 bp, outer-products = 297 bp; 3) rs33492148 forward inner primer (T allele specific) TTCTGTCTTAATTGAGCCCATATGAAAAT, reverse inner primer (G allele specific) GCACATTCTTCCAGACTCTGCATATC, forward outer primer ACTCTTGACAAAGAAGAATGCTTGCTTT, reverse out primer ATGTTTTGGCTAAGCACAATCCTACTCT, T-allele size = 194 bp, G-allele size = 172 bp, outer-products = 311 bp; 4) rs32348286 forward inner primer (A allele specific) AGGACTGCCACAGGCCAGCATCTCACA, reverse inner primer (G allele specific) AGTGTATCTATCAGGTGAATTCCAGTAGTC, forward outer primer AAGCCAAGCTGTCTCCAAGTCCTAGAAA, reverse out primer ACCACTTGAAGCCTGATTAAAATGTGCC, A-allele size = 213 bp, G-allele size = 175 bp, outer-products = 331 bp; 5) rs29524465 forward inner primer (A allele specific) CCTCTAATCTCCTGAGGATTGGAACA, reverse inner primer (G allele specific) TCATTTGGACACTAGAGCTTCTTCATTATC, forward outer primer TCGGAGAGACAGTTGTCTGTTAGGTTTA, reverse out primer GACAATGACGAAAAGACAAGTCACTTCT, T-allele size = 116 bp, G-allele size = 127 bp, outer-products = 187 bp; 6) rs33719947 forward inner primer (C allele specific) GTACATGTTCTTTTAAAATTATTAATCGAC, reverse inner primer (T allele specific) AGAAAAAGACACTCCTTGGAGCATGA, forward outer primer CTGTGATTTAAAGGCGTGCTAGTACTAC, reverse out primer GTGAGAGAGAGAGTCTGGGAATATTTCT, C-allele size = 177 bp, T-allele size = 157 bp, outer-products = 278 bp. Each of the 110 MF1 mice were genotyped for these markers by PCR and the products were separated by 4% agarose gel and visualized by ethidium bromide staining.

### Gene Expression Analysis

RNA extraction was performed on the liver tissue obtained from each animal at the time of sacrifice, using Qiagen's RNeasy kit (cat# 74104). For the gene expression measurements, Illumina's Mouse whole genome expression BeadChips (MouseRef-8-v1 Expression BeadChip) were used. All amplifications and hybridizations were performed according to Illumina's protocol by the Southern California Genome Consortium microarray core laboratory at UCLA. In brief, 100 ng of total RNA was first reverse transcribed to cDNA using Ambion cDNA synthesis kit AMIL1791 and then converted to cRNA and labeled with biotin. 800ng of biotinylated cRNA product is hybridized to prepared whole genome arrays and allowed to incubate overnight ( 16–20 hrs) at 55 degrees C. Arrays were washed then stained with Cy3 labeled streptavadin. Excess stain wass removed by washing and the arrays were dried and scanned on an Illumina BeadScan con-focal laser scanner.

Data normalization was performed using the rank invariant method by the Bead Studio software. After normalization, all gene expression data were log2 transformed.

To filter genes, we selected probes which met two criteria; 1) the probes exhibited a reliable signal and 2) the probes contained no annotated SNP within their sequence. The former was determined according to the Illumina Bead Studio output. The detection value is equal to 1-probability that a signal level is due to nonspecific hybridization. This value can be interpreted as the probability of seeing a certain signal level without specific probe-target hybridization. For filtering we excluded any probe which had a detection value of lower than 0.95 in greater than eleven (10%) or more animals. To select against the bias in hybridization due to probe design, as described by Walter et al [40], we excluded any probe which in blast search had 100% sequence identity to more than one location of the genome or had at least one SNP within it. To determine this we aligned the genomic location of all the probes against the genomic location of ∼8 million SNPs available on the Perlegen database (http://mouse.perlegen.com/mouse/index.html). Any probe which contained a SNP which was polymorphic between the proposed ancestors of the MF1 mice (I,e. C57BL/6J, DBA/2J, C3H/HeJ, AKR/J, I/LnJ, BALB/cJ, RIII/J, and A/J) was excluded. This filtering step resulted in the exclusion of 2160 probes. The remaining 10013 probes were used as the starting set for the whole genome association analysis. The gene expression data are deposited to the Gene Expression Omnibus (http://www.ncbi.nlm.nih.gov/geo/) at NCBI. These data are deposited under the accession number GSE10280.

### Linkage Disequilibrium (LD) and False Discovery Rate (FDR) Calculation

LD and FDR were calculated using R software algorithms. To compute the pair-wise LD between markers, we used the LD function in the Genetics package, which includes output of the chi square p-values for marker independence which we used to test for LD between markers on different chromosomes. For the FDR calculation we used the q-value package in R [41]. Due to the computational complexity associated with evaluating q-values for >20 million p-values, we computed the FDRs by taking the average FDR for 100 samples each containing 5 million randomly selected p-values from the original 22,069,649 calculated p-values.

### Whole Genome Association and Population Substructure Correction

We first computed the genetic similarity matrix between the individual mice as the fraction of shared alleles (identity-by-state) for each pairs, and visualized it with heatmap R package. A complex multi-leveled population structure and genetic relatedness is observed in the genetic similarity matrix.

We applied the following variance component test to estimate the variance explained by genetic background and assess the statistical significance.
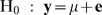


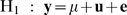
where y is the vector of expression values of a gene, and μ is mean, and e is random errors following an identical and independent normal distribution with Var(e) = σ_e_^2^I. u is a vector of random variables accounting for the effect from genetic background. u follows a multivariate normal distribution with Var(u) = σ_g_^2^K, where K is the genetic similarity matrix described above. The fraction of variance explained by genetic background is computed as previously suggested with tr(SKS)/(n-1+tr(SKS)), where S = I-J/n, and J is a square matrix consisting of ones from the REML estimate of H_1_. The likelihood ratio test is performed by comparing the maximum likelihood of two hypotheses. The likelihood difference 2*(*l_1_-l_0_*) asymptotically follows a 1:1 mixture of the chi-squared distribution with zero and one degree-of-freedom [42]. The false discovery rate is estimated conservatively by setting π_0_ = 1 [41].

To account for population structure and genetic relatedness in association mapping, we applied the following standard linear mixed model as previously suggested [18],[35],[36].
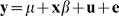
where y, μ, u, and e are same as described above, and x is the genotype vector of a marker represented in additive model, and β is a marker effect. A standard F test was performed to test H_1_:β≠0 against H_0_:β = 0 after estimating restricted maximum likelihood (REML) variance components as described [18],[35],[36]. We applied EMMA (Efficient Mixed Model Association) as a R implementation of linear mixed model. Since EMMA is orders of magnitude faster than other implementations commonly used, we were able to perform statistical analyses for all pairs of transcripts and genome wide markers in a few hours using a cluster of 50 processors.

## Supporting Information

Figure S1 LD structure on Chromosome 2-20. The order of markers in each heat map follows the physical location of the marker along the chromosome with the most proximal at the bottom and the most distal marker on the top. The correspondence between color and r^2^ is shown in the insert.
(0.23 MB DOC)

Figure S2 Effect of familial structure on gene expression association. Panel (A) shows the inflation of false positives at a transcript represented by as the average log p values across all the markers (x-axis) and the correlation between a transcript and genetic relatedness (y-axis). Panel (B) shows this correspondence after correcting for genetic relatedness using a linear mixed model.
(4.23 MB DOC)

Figure S3 Power Analysis. Figures A–E show the power expected for various various genetic backgrounds. A is for no genetic background effect, B is for genetic background effect of 0.1, C is for genetic background effect of 0.2, D is for genetic background effect of 0.4, and E is for genetic background effect of 0.5. In each panel, the power is calculated for various p-value cutoffs (grey = 0.05, green = 0.01, orange = 0.001, blue = 0.0001, red = 1e-05, purple = 1e-06, black = 2.76e-05 which is equivalent to the Bonferroni correction). For each calculation, the minor allele frequency is assumed 0.3.
(0.08 MB DOC)

Figure S4 Validation of distant eQTL hotspots. In each of the figures A through D, the 110 mice were randomly split two groups (55 MF1 mice in each) and for each subset the number of distant eQTL counts were determined across the genome. The genome is represented as 1287 equally sized bins of 2 Mb. The gray line depicts the 0.05 genome wide significance for eQTL enrichment after Bonferroni correction (p-value of 3.9e-05).
(0.08 MB DOC)

Figure S5 Association results in MF1 data for 14 distal eQTLs co-localized in the BxH F2 intercross. The location of local eQTLs Pparg (grey), Cidec (red), and Bcl2l13 (black) is shown at the bottom of each figure.
(0.13 MB DOC)

Table S1 *Cis* and *trans* eQTL at 1%, 5%, 10%, and 25% FDR.
(1.17 MB XLS)
