# Supplementary material for: High-Resolution Mapping of Gene Expression Using Association in an Outbred Mouse Stock
Source: PLoS Genet. 2008 Aug 8;4(8):e1000149. doi: 10.1371/journal.pgen.1000149 (PMC2483929; doi:10.1371/journal.pgen.1000149)
Supplement: Figure S1 — LD structure on Chromosome 2-20. The order of markers in each heat map follows the physical location of the marker along the chromosome with the most proximal at the bottom and the most distal marker on the top. The correspondence between color and r2 is shown in the insert. (0.23 MB DOC) [file pgen.1000149.s001.doc]

Supplemental Figure 1. LD structure on Chromosome 2-20. The order of markers in each heat map follows the physical location of the marker along the chromosome with the most proximal at the bottom and the most distal marker on the top. The correspondence between color and r^2^ is shown in the insert.

Chromosome 2

Chromosome 3

Chromosome 4


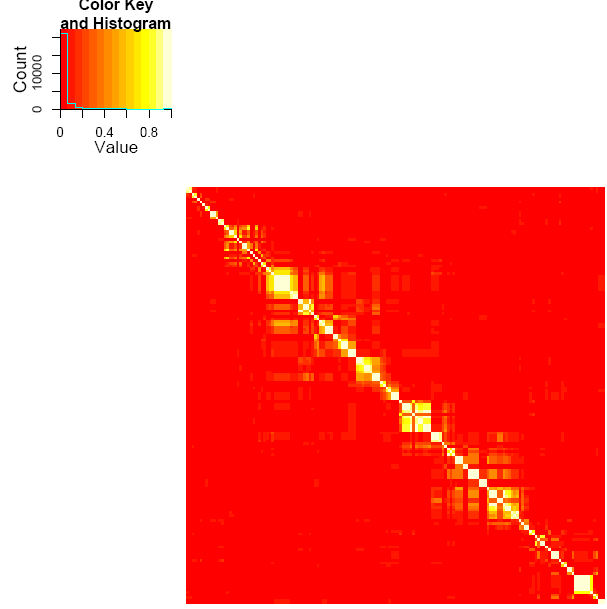


Chromosome 5

Chromosome 6

Chromosome 7

Chromosome 8

Chromosome 9

Chromosome 10

Chromosome 11

Chromosome 12

Chromosome 13

Chromosome 14

Chromosome 15

Chromosome 16

Chromosome 17

Chromosome 18

Chromosome 19

Chromosome X
