# Supplementary material for: High-Resolution Mapping of Gene Expression Using Association in an Outbred Mouse Stock
Source: PLoS Genet. 2008 Aug 8;4(8):e1000149. doi: 10.1371/journal.pgen.1000149 (PMC2483929; doi:10.1371/journal.pgen.1000149)

Supplemental Figure 2. Effect of familial structure on gene expression association. Panel (A) shows the inflation of false positives at a transcript represented by as the average log p values across all the markers (x-axis) and the correlation between a transcript and genetic relatedness (y-axis). Panel (B) shows this correspondence after correcting for genetic relatedness using a linear mixed model.

A)


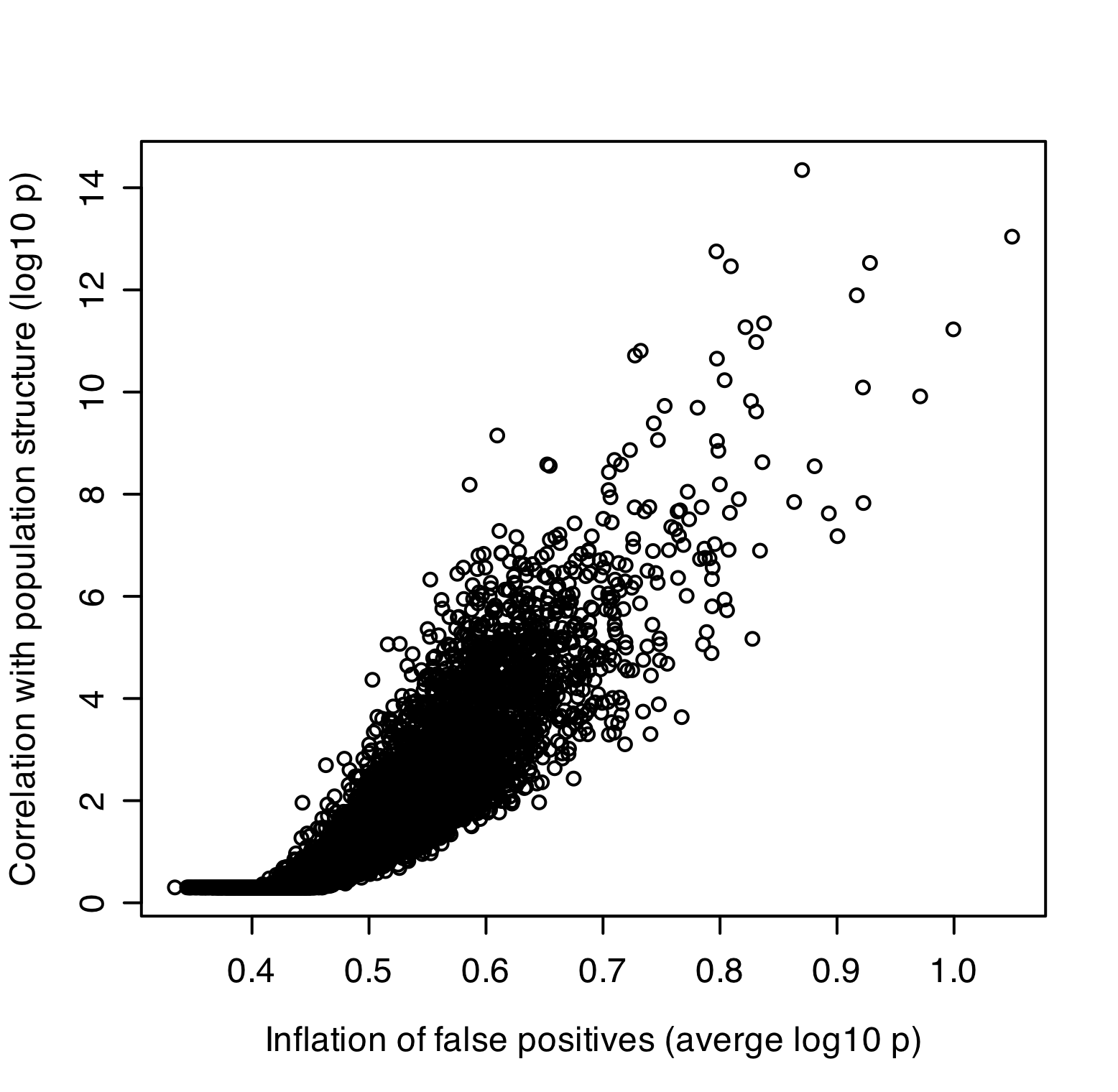


B)


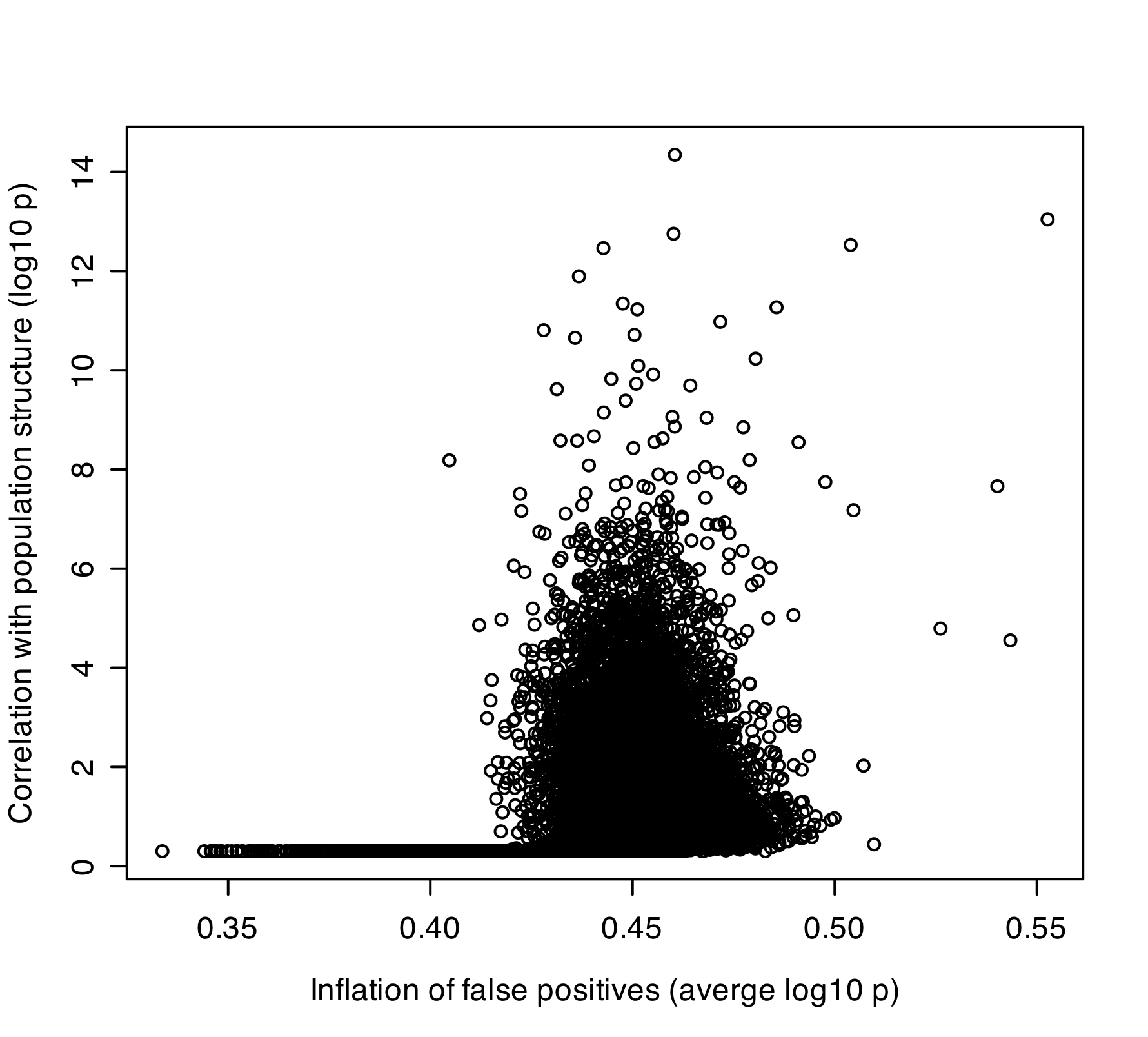

Supplement: Figure S2 — Effect of familial structure on gene expression association. Panel (A) shows the inflation of false positives at a transcript represented by as the average log p values across all the markers (x-axis) and the correlation between a transcript and genetic relatedness (y-axis). Panel (B) shows this correspondence after correcting for genetic relatedness using a linear mixed model. (4.23 MB DOC) [file pgen.1000149.s002.doc]
