# Supplementary material for: High-Resolution Mapping of Gene Expression Using Association in an Outbred Mouse Stock
Source: PLoS Genet. 2008 Aug 8;4(8):e1000149. doi: 10.1371/journal.pgen.1000149 (PMC2483929; doi:10.1371/journal.pgen.1000149)

Supplemental Figure 3. Power Analysis. Figures A-E show the power expected for various various genetic backgrounds. A is for no genetic background effect, B is for genetic background effect of 0.1, C is for genetic background effect of 0.2, D is for genetic background effect of 0.4, and E is for genetic background effect of 0.5. In each panel, the power is calculated for various p-value cutoffs (grey=0.05, green=0.01, orange=0.001, blue=0.0001, red=1e-05, purple=1e-06, black=2.76e-05 which is equivalent to the Bonferroni correction). For each calculation, the minor allele frequency is assumed 0.3.

A)


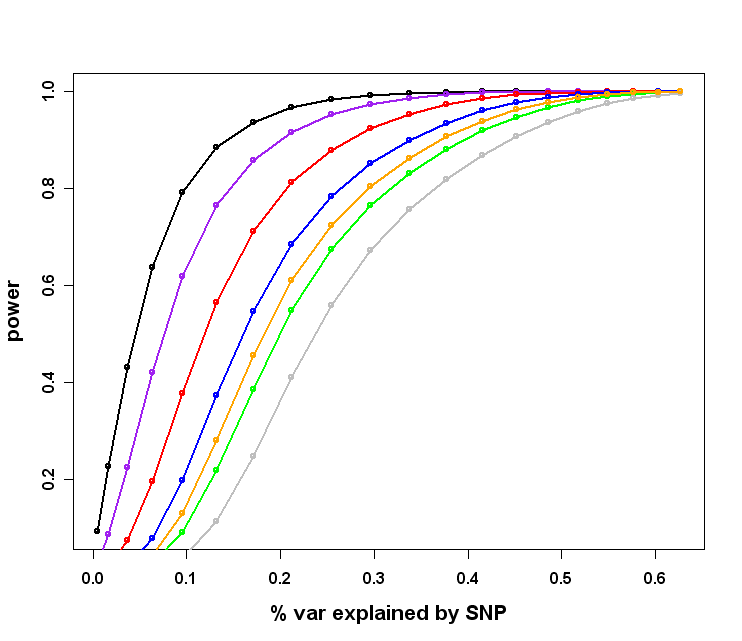


B)


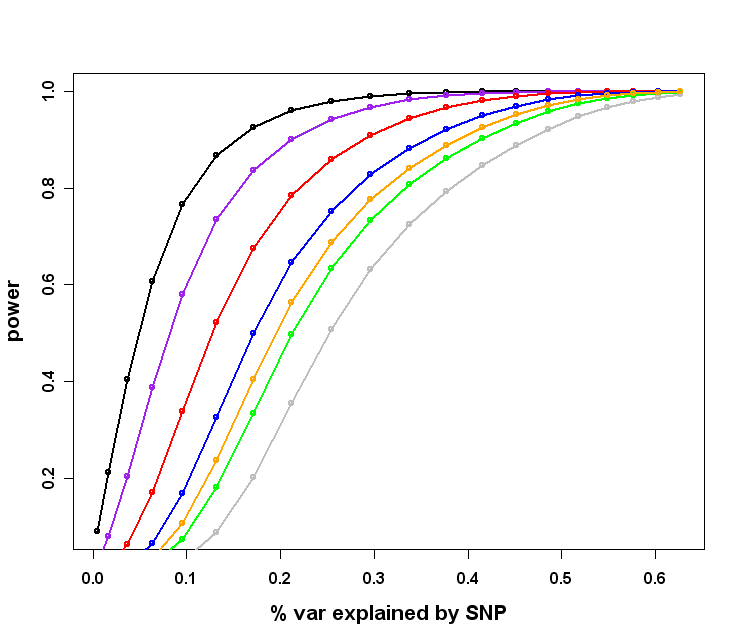


C)


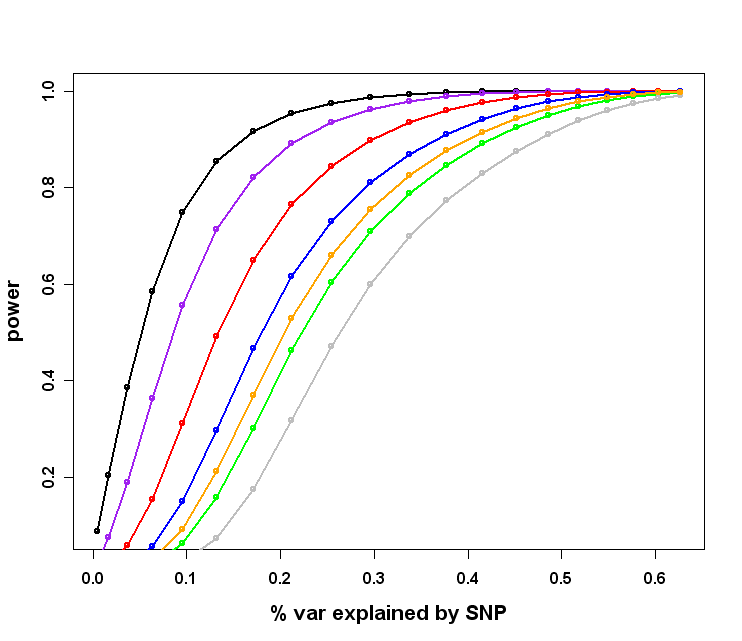


D)


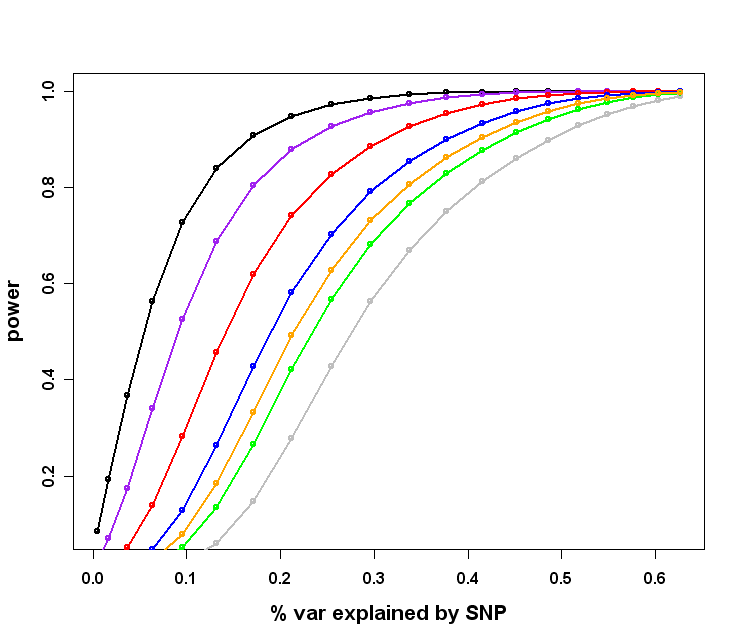


E)


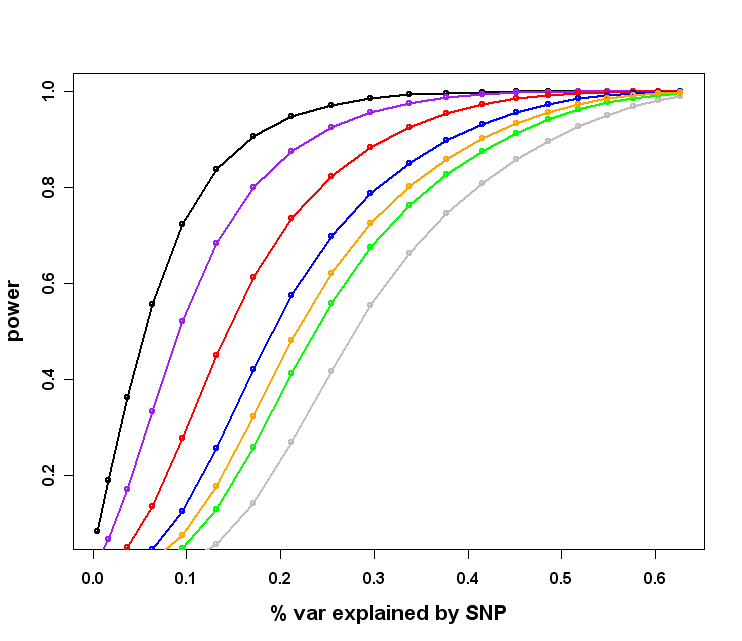

Supplement: Figure S3 — Power Analysis. Figures A–E show the power expected for various various genetic backgrounds. A is for no genetic background effect, B is for genetic background effect of 0.1, C is for genetic background effect of 0.2, D is for genetic background effect of 0.4, and E is for genetic background effect of 0.5. In each panel, the power is calculated for various p-value cutoffs (grey = 0.05, green = 0.01, orange = 0.001, blue = 0.0001, red = 1e-05, purple = 1e-06, black = 2.76e-05 which is equivalent to the Bonferroni correction). For each calculation, the minor allele frequency is assumed 0.3. (0.08 MB DOC) [file pgen.1000149.s003.doc]
