# Supplementary material for: High-Resolution Mapping of Gene Expression Using Association in an Outbred Mouse Stock
Source: PLoS Genet. 2008 Aug 8;4(8):e1000149. doi: 10.1371/journal.pgen.1000149 (PMC2483929; doi:10.1371/journal.pgen.1000149)

Supplemental Figure 4. Validation of distant eQTL hotspots. In each of the figures A through D, the 110 mice were randomly split two groups (55 MF1 mice in each) and for each subset the number of distant eQTL counts were determined across the genome. The genome is represented as 1287 equally sized bins of 2 Mb. The gray line depicts the 0.05 genome wide significance for eQTL enrichment after Bonferroni correction (p-value of 3.9e-05).

A)


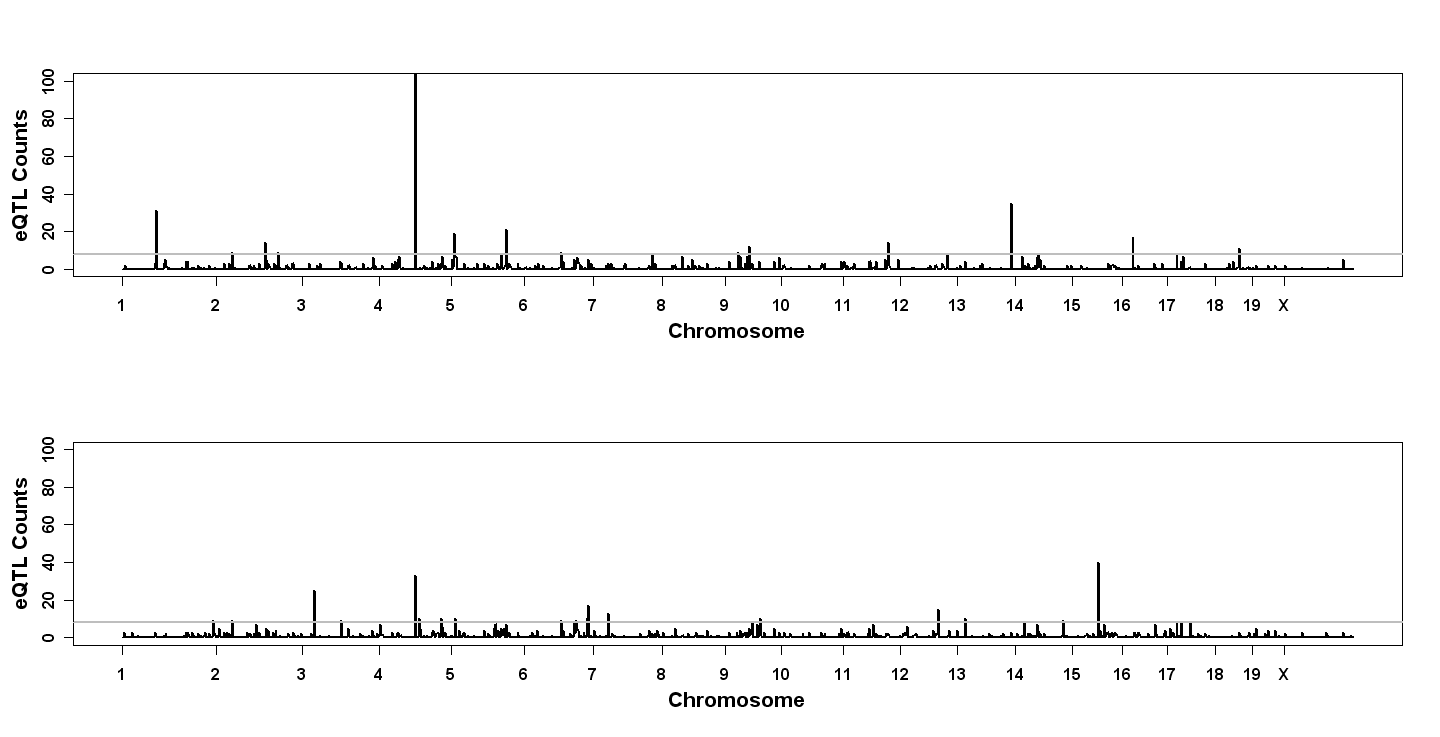


B)


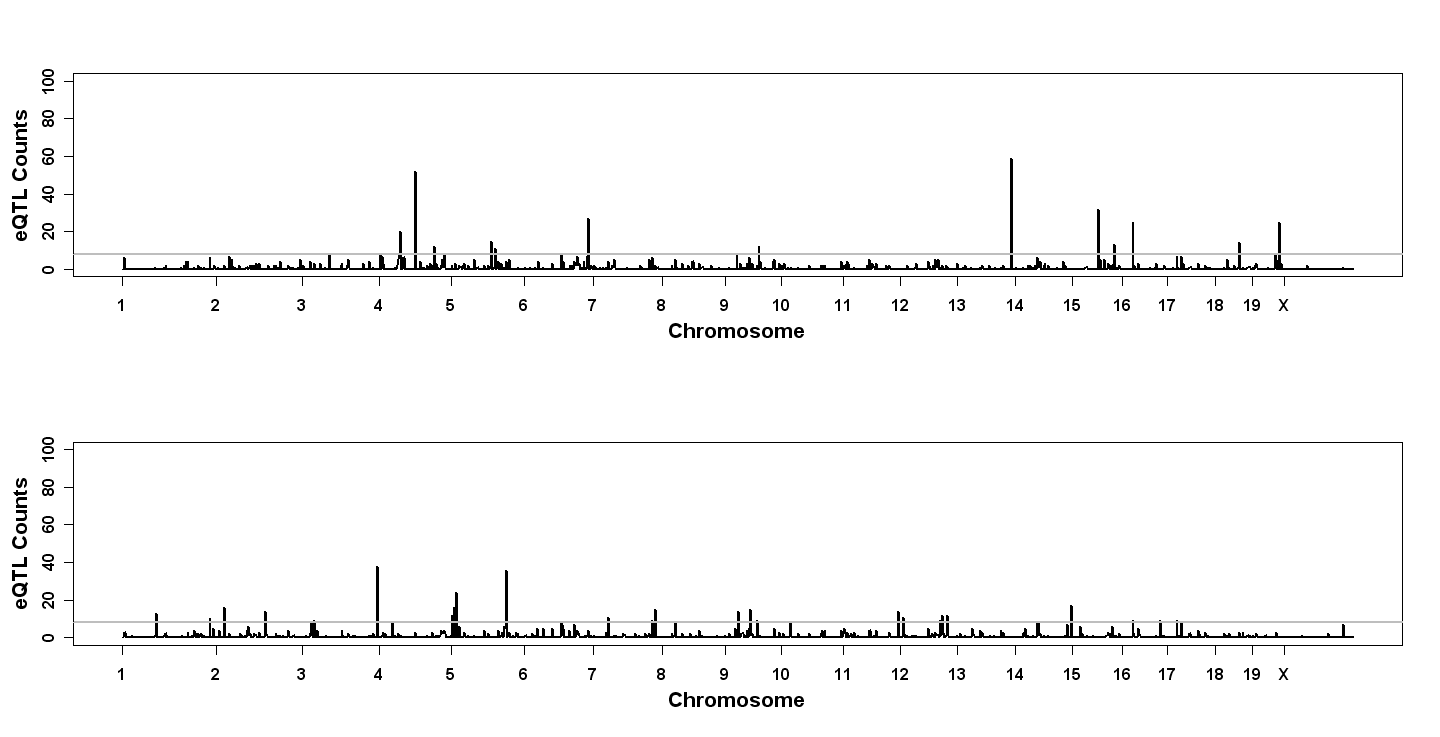


C)


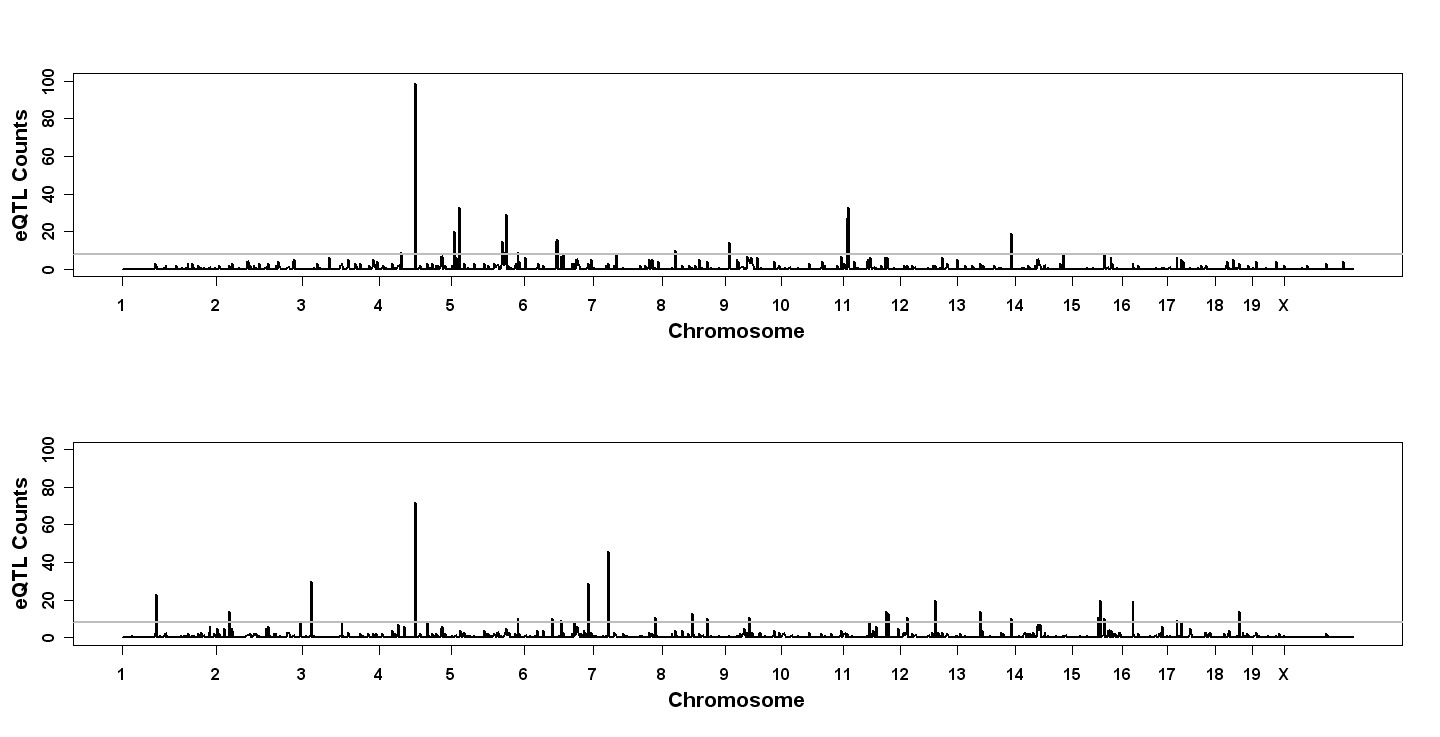


D)


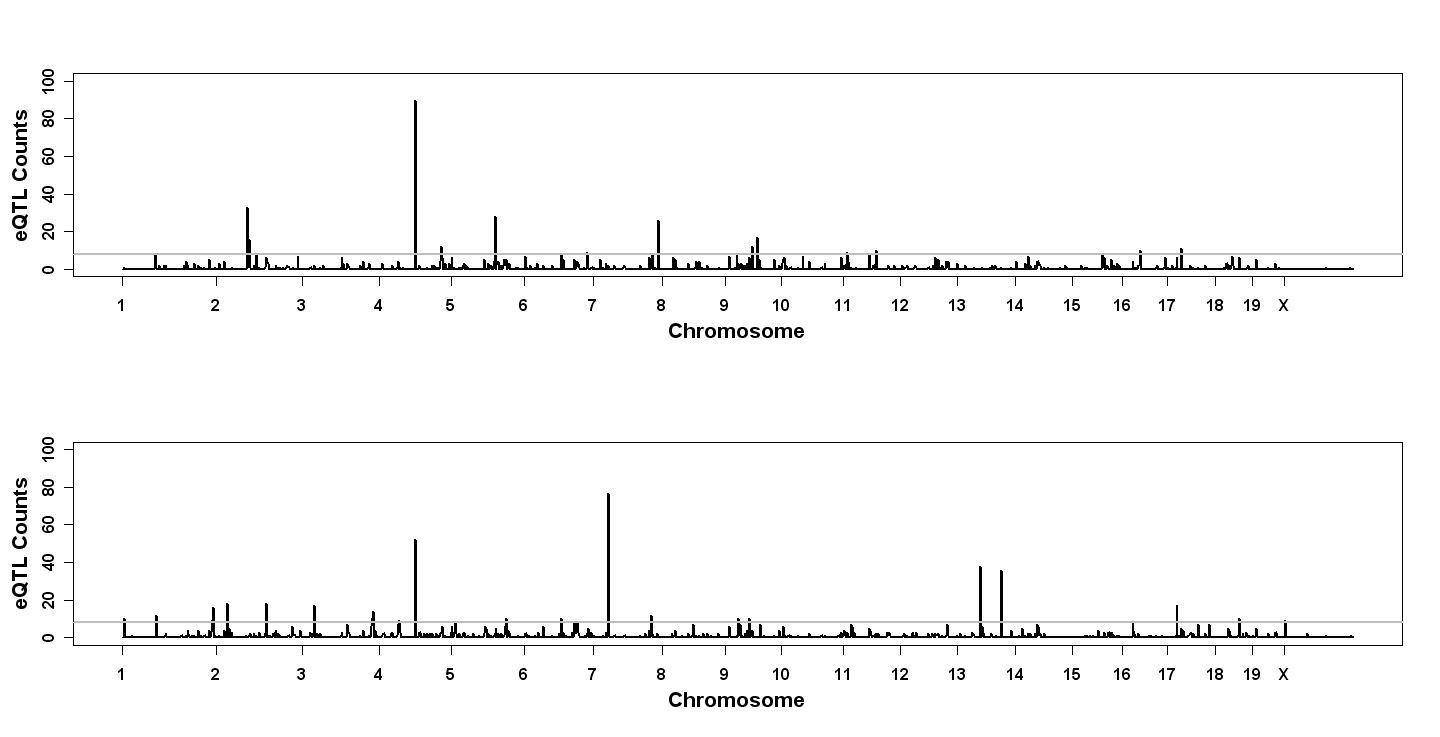

Supplement: Figure S4 — Validation of distant eQTL hotspots. In each of the figures A through D, the 110 mice were randomly split two groups (55 MF1 mice in each) and for each subset the number of distant eQTL counts were determined across the genome. The genome is represented as 1287 equally sized bins of 2 Mb. The gray line depicts the 0.05 genome wide significance for eQTL enrichment after Bonferroni correction (p-value of 3.9e-05). (0.08 MB DOC) [file pgen.1000149.s004.doc]
