# Supplementary material for: High-Resolution Mapping of Gene Expression Using Association in an Outbred Mouse Stock
Source: PLoS Genet. 2008 Aug 8;4(8):e1000149. doi: 10.1371/journal.pgen.1000149 (PMC2483929; doi:10.1371/journal.pgen.1000149)

Supplemental Figure 5. Association results in MF1 data for 14 distal eQTLs co-localized in the BxH F2 intercross. The location of local eQTLs Pparg (grey), Cidec (red), and Bcl2l13 (black) is shown at the bottom of each figure.


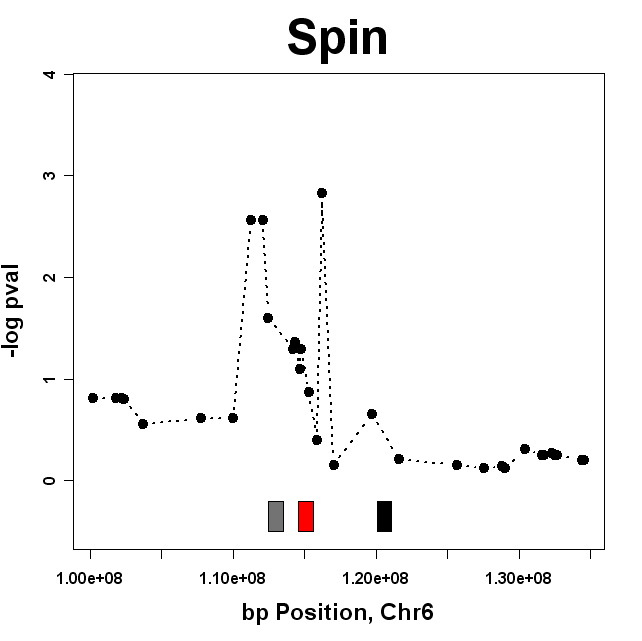


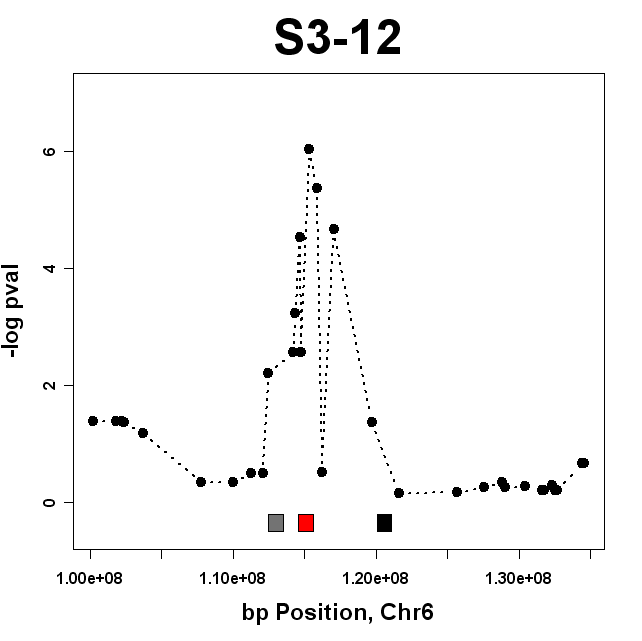


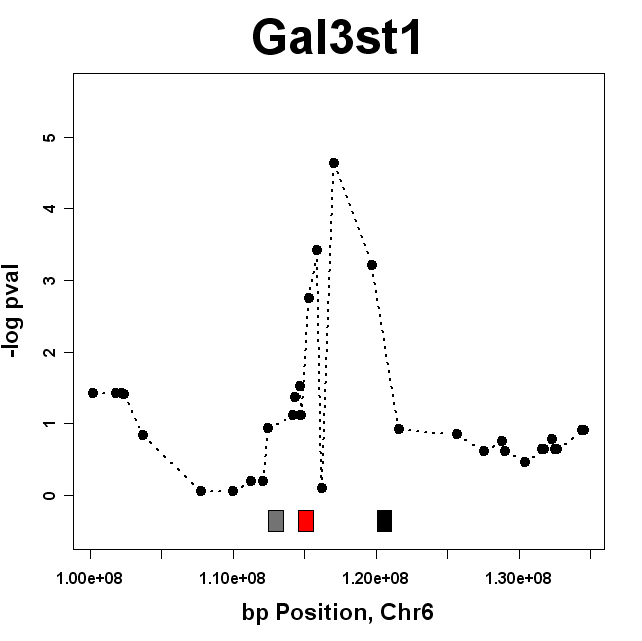


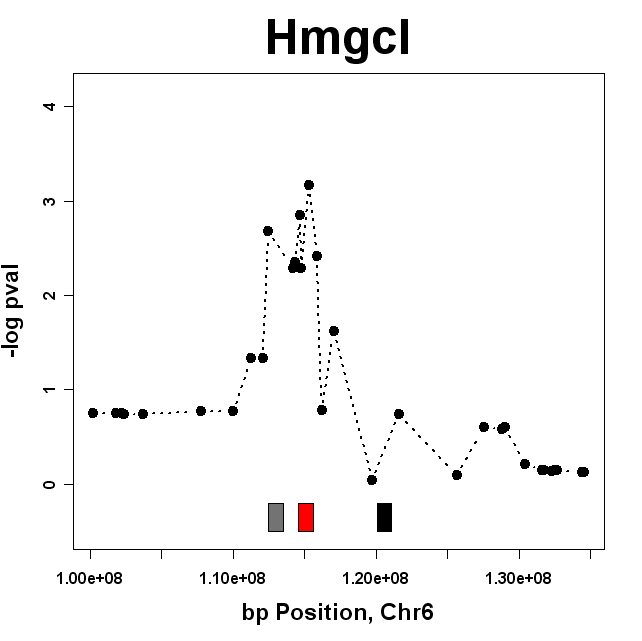


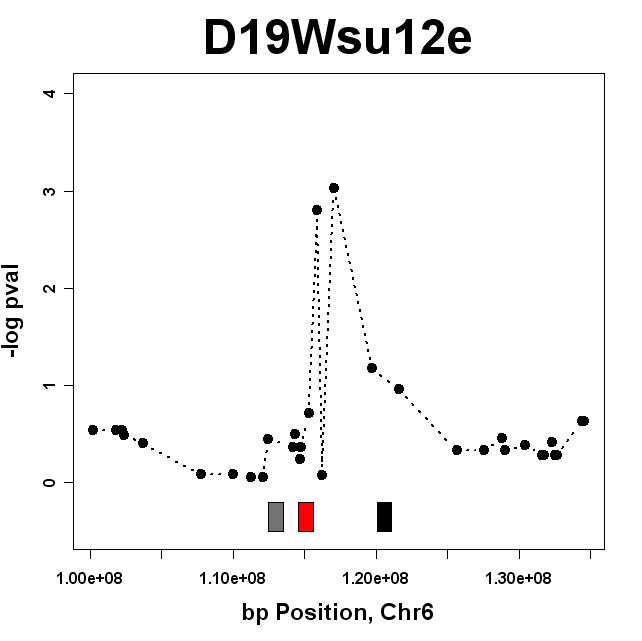


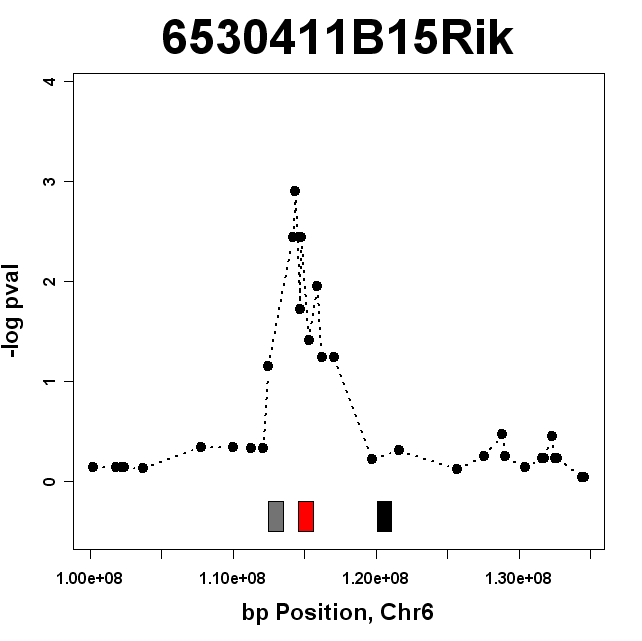


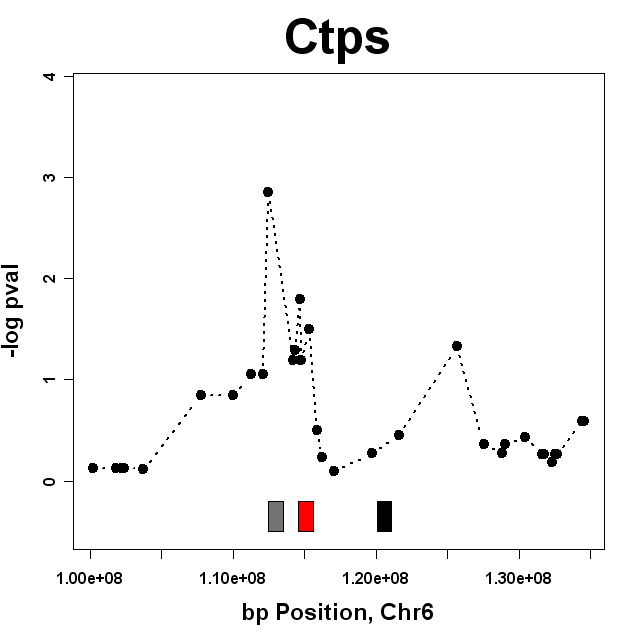


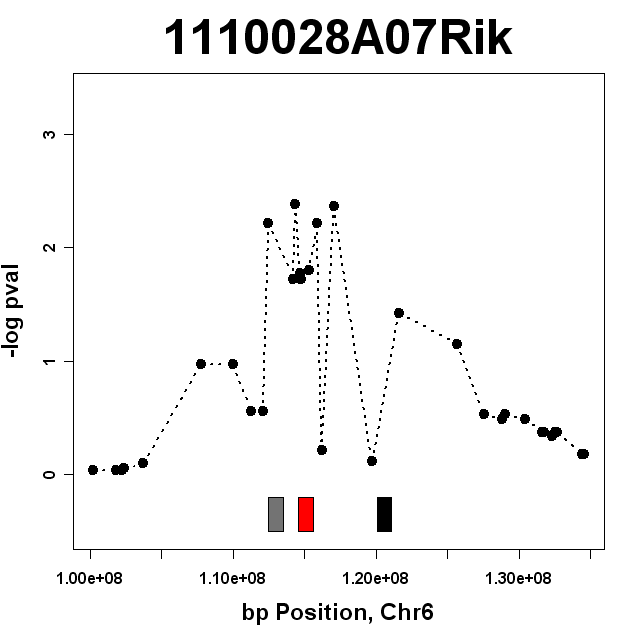


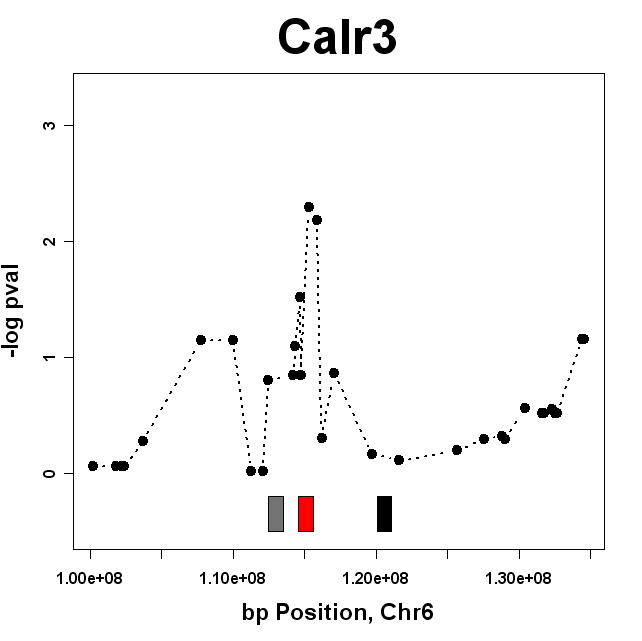


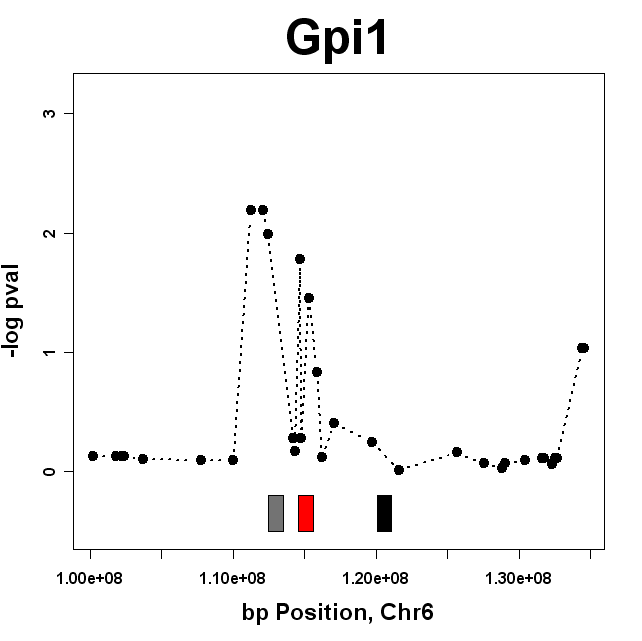


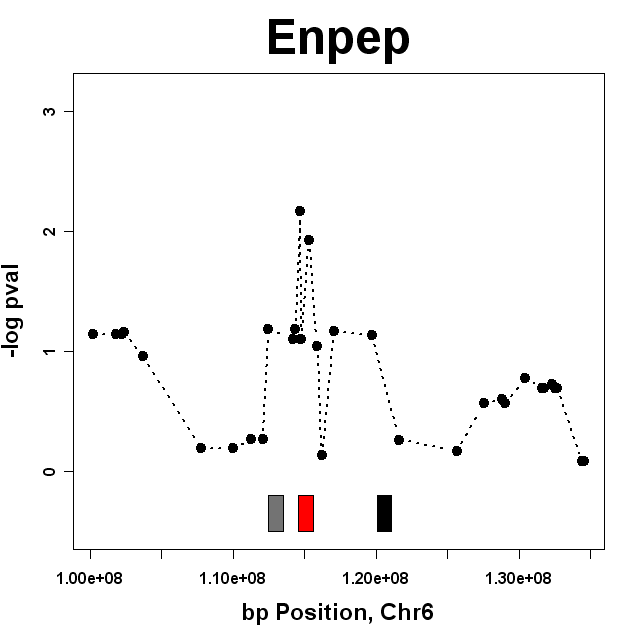


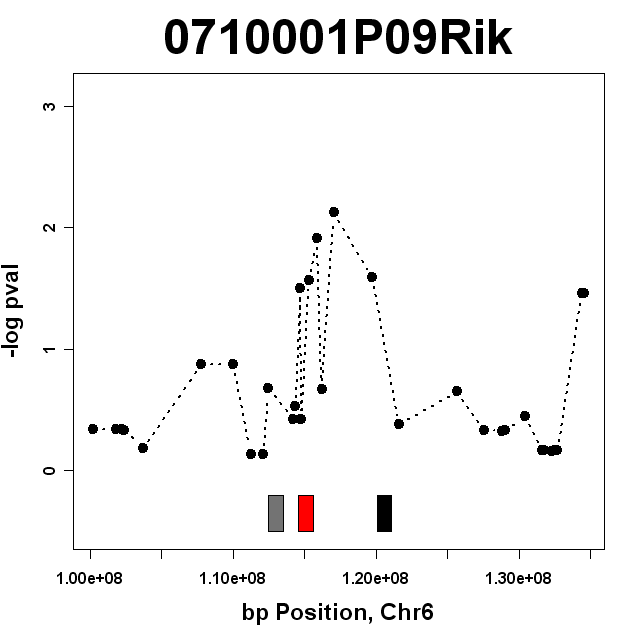


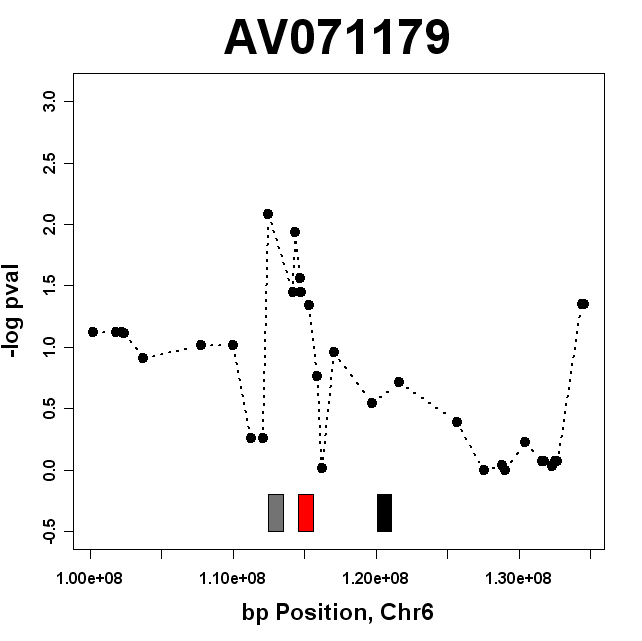


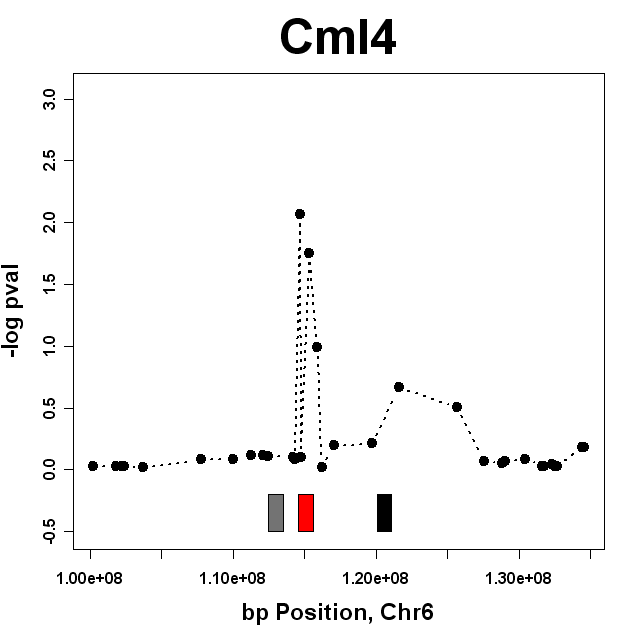

Supplement: Figure S5 — Association results in MF1 data for 14 distal eQTLs co-localized in the BxH F2 intercross. The location of local eQTLs Pparg (grey), Cidec (red), and Bcl2l13 (black) is shown at the bottom of each figure. (0.13 MB DOC) [file pgen.1000149.s005.doc]
